# Ontogeny of Unstable Chromosomes Generated by Telomere Error in Budding Yeast

**DOI:** 10.1371/journal.pgen.1006345

**Published:** 2016-10-07

**Authors:** Tracey Beyer, Ted Weinert

**Affiliations:** Department of Molecular and Cellular Biology, University of Arizona, Tucson, Arizona, United States of America; Columbia University, UNITED STATES

## Abstract

DNA replication errors at certain sites in the genome initiate chromosome instability that ultimately leads to stable genomic rearrangements. Where instability begins is often unclear. And, early instability may form unstable chromosome intermediates whose transient nature also hinders mechanistic understanding. We report here a budding yeast model that reveals the genetic ontogeny of genome rearrangements, from initial replication error to unstable chromosome formation to their resolution. Remarkably, the initial error often arises in or near the telomere, and frequently forms unstable chromosomes. Early unstable chromosomes may then resolve to an internal "collection site" where a dicentric forms and resolves to an isochromosome (other outcomes are possible at each step). The initial telomere-proximal unstable chromosome is increased in mutants in telomerase subunits, Tel1, and even Rad9, with no known telomere-specific function. Defects in Tel1 and in Rrm3, a checkpoint protein kinase with a role in telomere maintenance and a DNA helicase, respectively, synergize dramatically to generate unstable chromosomes, further illustrating the consequence of replication error in the telomere. Collectively, our results suggest telomeric replication errors may be a common cause of seemingly unrelated genomic rearrangements located hundreds of kilobases away.

## Author Summary

Genomic instability forms unstable chromosomes that generate genomic rearrangements associated with human disease. Because unstable chromosomes are inherently dynamic and rarely observed, mechanisms of instability are often inferred from genomic sequencing of the end state. Longitudinal observation of events, from initiation to resolution to a stable state, is rarely feasible. Here we document DNA replication errors at the chromosome end that lead to the formation of unstable chromosomes; the unstable chromosomes progressively rearrange and then resolve to stable structures. Error between DNA replication and telomere maintenance synergize to form unstable chromosomes at an extremely high frequency. Surprisingly, we find unstable chromosomes often convert to a stable form in a single centromeric region we previously suggested was a fragile site, that we now call a "collection site". Thus, the most commonly recovered end state of chromosome instability, which localizes to the middle of the chromosome, is caused by replication error at the chromosome end. Our findings are relevant to mechanisms impacting short-term (pathology) and long-term evolution, including genomic instability, telomere replication error and events at fragile sites.

## Introduction

Faithful replication of the genome prevents chromosome instability. Replication error causing chromosome instability results in a plethora of changes, including deletion, insertion, translocations, and loss. The multi-protein DNA replication complex, called the replisome, undergoes still untold changes, with unknown consequences, when it encounters difficulties (e.g. DNA damage, replication fork blocking proteins, and repetitive sequences). The replisome may slow, or stop and/or restart synthesis, all of which can be detrimental and give rise to genomic changes ([1–5] for review).

Some regions of the genome are particularly prone to replication error [6–9]; the telomere is one such difficult region [10–14]. How the telomere disrupts replication is still a matter of debate. Disruption to telomere replication may occur due to the repetitive nature of telomere sequences, secondary structures, chromatin complex, or to complications of terminal replication [10,15–17]. In addition to replication error, telomere loss may be caused by telomerase deficiency or resection of uncapped telomeres [18–21]. Integrity of the protective end is critical to chromosome maintenance [22–24], and loss of telomere sequence and/or telomere binding proteins renders the telomere prone to rearrangement [25–33].

Complicating the study of replication errors is that errors arising in the telomere, or elsewhere in the genome, frequently form inherently unstable chromosomes [34–36]. An unstable chromosome is dynamic, beginning as a single rearrangement from which multiple additional rearrangements emerge. Dicentric chromosomes, a single chromosome with two centromeres, tend to be highly unstable owing to mitotic segregation error [37–41]. Dicentrics can undergo successive changes, including the formation of de novo dicentrics [22,42–45]. Unstable chromosomes can take on other forms aside from dicentrics, though those are less well-defined [46–51]. The transient nature of unstable chromosomes renders them difficult to study. In fact, in an earlier study of telomerase defects and instability, unstable chromosomes were not detected [27,28].

Here we investigate the ontogeny of events that form unstable chromosomes in budding yeast, from initiation to resolution to stability. We find that instability can initiate by replication error in the telomere, and frequently resolves to the middle of the chromosome, which we call a “collection site”. Telomerase and *tel1Δ* mutants, each with telomere-specific roles, induce a high frequency of unstable chromosomes. Further, a *tel1Δ* mutation synergizes with an *rrm3Δ* mutation, defective in the DNA helicase, to form unstable chromosomes at an extremely high frequency (> 1 in 100 cells). We infer that even in *rad9Δ* mutants, with no telomere-specific function, instability begins in or near the telomere. Once formed, physically longer unstable chromosomes progress to the physically shorter unstable chromosomes, including a specific dicentric studied previously [35,52]. We infer that events initiate by replication error in or near the telomere, and then progress to other regions of the chromosome, a process that we suggest is relevant to genomic rearrangements that are seemingly unrelated to error at the telomere.

## Results

### The model system

**Fig 1:**
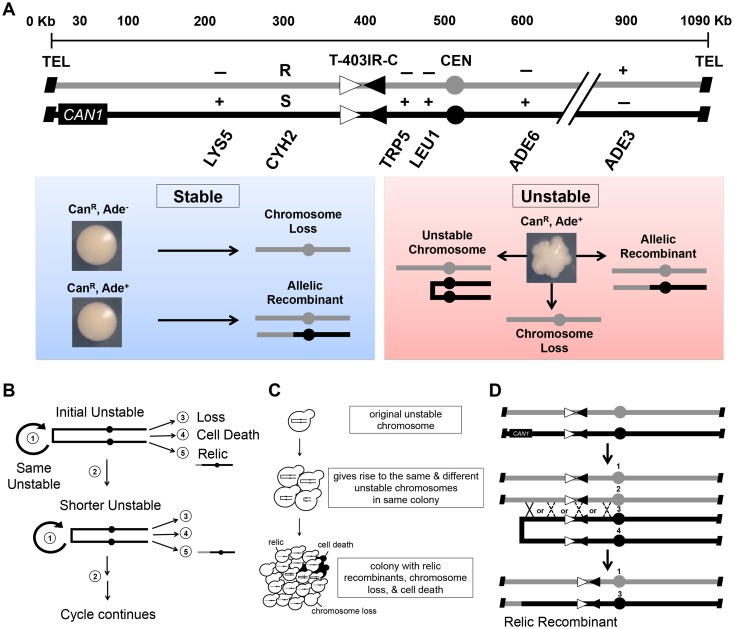
The Chr VII disome system detects unstable chromosomes. (A) Diagrams of Chr VII homologs in the starting strain (one gray, one black). Genetic markers are labeled in their approximate locations (scale above homologs); a (+) or (-) indicates the functional status of each allele. In the T-403IR-C site inverted repeats: open and black arrowheads are Ty3 LTR sigma 2 and sigma 3, respectively (each is ~200 bp); telomeres: black diagonal rectangles; centromeres: gray or black circles. Blue and red boxes: Can^R^ round colonies with stable chromosome changes (blue box) or Can^R^ sectored colonies formed by an unstable chromosome and forming multiple chromosome changes (red box). Gray and black lines represent Chr VII sequences from each homolog. (B) The five possible fates of unstable chromosomes: 1) the same unstable chromosome is inherited by the daughter cell, 2) further change to form a different unstable chromosome, 3) loss, 4) cell death, or 5) allelic recombination to form a “relic recombinant.” (C) How genetic diversity may arise in a sectored colony. See text for explanation. Changes to Chr VII are shown in cells; shaded indicates cell death, absence of a Chr VII indicates loss. (D) How relic recombinants arise. The initial chromosomes (top) replicate with error, forming a G2 cell with fused sisters of the bottom homolog. That unstable chromosome breaks, and undergoes recombination with the homolog (denoted by X’s; solid line for this example, dashed lines are other possible options). Co-segregation of #1 and #3 forms a stable relic recombinant (labeled below). The unstable chromosome may instead undergo complex reactions to form an isochromosome (not depicted).

In this study we use a budding yeast genetic system shown in Fig 1A and reported previously [35,52]. The yeast strain is a haploid that contains two homologs of Chr VII (a Chr VII disome). The Chr VII homologs are extensively genetically marked, as indicated, to assist in genetic analyses of spontaneous chromosomes changes.

Chromosome changes are rare, necessitating genetic selection to detect. We use the *CAN1* gene in a negative selection scheme to detect all changes, whose frequencies occur at between 1 in 10^5^ to 1 in 10^2^ cells, depending on event and mutant background. Using the Chr VII disome, we detect three distinct chromosome changes: unstable chromosomes, allelic recombination, and chromosome loss. We detect chromosome changes by selecting first for loss of *CAN1*, and then analyzing progeny. *CAN1* encodes arginine permease that allows uptake of canavanine, a toxic analog of arginine. Cells with unchanged chromosomes retain *CAN1* and die on media containing canavanine (Can^S^), while cells that lose *CAN1* become canavanine resistant (Can^R^).

To detect chromosome instability, the assay proceeds as follows: we grow cells that initially have intact Chr VIIs on rich media for approximately 20 generations, during which chromosome changes occur (S1 Fig). We then select for cells with chromosome changes by plating to selective solid media. Can^R^ colonies form in 2 to 5 days.

Can^R^ colony morphology and genotype indicate chromosome changes. Allelic recombinants and chromosome loss events generate round colonies, while unstable chromosomes form sectored colonies (Fig 1A). Round colonies are either Can^R^ Ade^-^, formed by a chromosome loss event, or Can^R^ Ade^+^, formed by an allelic recombination event. Importantly, round colonies have the expected property that most cells (> 95%) taken from a round colony have the same phenotype (see Methods; Fig 1A for one example). Because most cells taken from a round colony have the same phenotype, we infer that the first Can^R^ cell on the selective plate had a stable karyotype inherited without further change in the progeny (in that given colony).

Strikingly, many Can^R^ Ade^+^ colonies are sectored instead of round (e.g. *rad9Δ* checkpoint mutants have a 6-fold increase in the frequency of sectored colonies, 91 x 10^−5^, relative to the frequency of round colonies, 15 x 10^−5^), and these prove to be generated by unstable chromosomes (Fig 1A). Sectored colonies have the unexpected property that individual cells taken from the colony frequently have *different* phenotypes. Because cells taken from a sectored colony have different phenotypes, we infer that the first Can^R^ cell on the selective plate had an *unstable* karyotype, inherited with further changes in the progeny (Fig 1B and 1C). Sectoring is due to a combination of poor growth, cell death, and chromosome changes as cells grow on the selective plate. A hypothetical example of how one unstable chromosome may give rise to different chromosomes is shown in Fig 1B; the unstable chromosome can form more of itself, form other unstable chromosomes, be lost, cause cell death, or recombine with the intact homolog to yield a recombinant.

### Allelic and relic recombinants

There are two types of related recombinants amongst Can^R^ Ade^+^ cells: allelic and relic recombinants. We use the term "allelic recombinant" to indicate a Can^R^ Ade^+^ cell that formed before plating cells onto selective media, where it formed a round colony with a stable karyotype. We use the term "relic recombinant" to indicate a Can^R^ Ade^+^ cell that formed in a sectored colony, as an unstable chromosome divides on selective media.

Allelic and relic recombinants have similar structures but distinct ontogenies. As a working model, we propose that an initial error occurs. That error leads to two possible outcomes: either allelic recombination or an unstable chromosome that subsequently leads to relic recombinants (S2 Fig). Allelic recombinants may arise from the initial replication error by recombination with the intact homolog, to directly form a stable Can^R^ Ade^+^ chromosome. In contrast, relic recombinants arise after an unstable chromosome forms by repair between sister chromatids, and that unstable chromosome then recombines with the homolog to resolve to a stable relic recombinant (Fig 1D and S2 Fig). In support of their distinct ontogenies, we find that Can^R^ Ade^+^ allelic and relic recombinants give different distribution profiles in wild type, *rad9Δ*, and *tel1Δ* cells; allelic recombinants most often form near the chromosome end and relic recombinants most often form near the middle of the chromosome (S2 Fig).

Relic recombinants are critical to our analysis: they give insight into the initial unstable chromosome structure. For example, if a sectored colony forms an unstable chromosome near the telomere, then we expect to recover relic recombinants with a telomere-proximal marker. Alternatively, if a sectored colony forms only centromere-proximal unstable chromosomes, then we do not expect to recover relic recombinants with a telomere-proximal marker, but rather only with centromere-proximal markers. In addition to the common relic recombinants shown in Fig 1D, we also detect a relic that is a previously analyzed isochromosome (discussed later) [35,52].

### Unstable chromosomes do not initiate in a "fragile site" near the centromere

**Fig 2:**
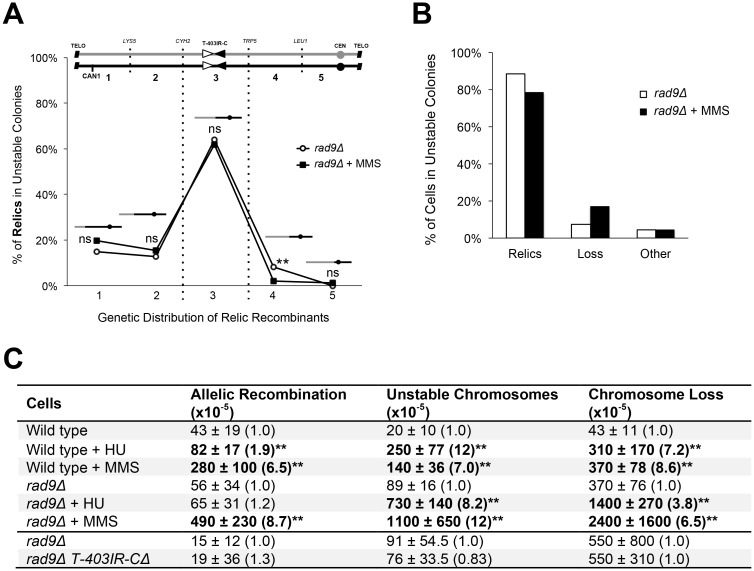
Unstable chromosomes resolve to the T-403IR-C region. (A) Relic recombinants can form in any genetic interval (labeled 1 through 5). Graph below shows the distribution of relics in *rad9Δ* Can^R^ Ade^+^ sectored colonies generated by growth with or without a 6 hr MMS exposure. N = 387 for untreated, and N = 260 for MMS-treated. Each percent value represents the percentage of relic recombinants in a particular interval. Statistically significant differences between untreated and MMS-treated relic recombinants are shown above each genetic interval (**P < 0.01 or non-significant (ns), Z score test for population proportions). (B) Distributions of relic recombinants, loss and “other” from *rad9Δ* Can^R^ Ade^+^ sectored colonies. N = 438 for untreated, and N = 332 for MMS-treated. Other: complex genotypes (i.e.: some cells in a sectored colony are still unstable, forming a colony on rich media that, upon replica plating, is half loss and half a relic recombinant.) (C) Frequency of the three instability events in wild type and *rad9Δ* mutants. Average frequency ± standard deviation shown. Fold changes are in parentheses and are relative to the same strain without drug treatment, or the T-403IR-C site deletion (*rad9Δ T-403IR-CΔ*) relative to *rad9Δ*. Statistically significant differences are in bold (**P < 0.01, Kruskal Wallis test).

Our previous studies suggested that replication error initiates events at a specific site we call "T-403IR-C" (for “Telomere—403 Kb Inverted Repeat—Centromere”), a site four-fifths of the way towards the centromere (Fig 1A, [35,52]). This ~4 Kb site has an intriguing structure, suggesting it disrupts replication, and includes inverted repeats that fuse to form a dicentric [52]. The T-403IR-C site is present in a larger genetically defined 94 Kb region, bordered by *CYH2* and *TRP5*. Sectored colonies arising from unstable chromosomes, generate relic recombinants enriched in the larger 94 Kb region (in both wild type cells and in *rad9Δ* mutants; [52] and Fig 2A and S2 Fig). In sum, we expected that the T-403IR-C site was a fragile site where events often initiate.

Several arguments and observations suggest, however, that initial events that form unstable chromosomes rarely start in the T-403IR-C site. First, if allelic and unstable chromosomes have a common origin, then the fact that many allelic recombinants in wild type cells and in *rad9Δ* cells (and in *tel1Δ* mutants, studied further below) form in a telomere-proximal genetic interval argues that the T-403IR-C region is not a common site of initial error for unstable chromosomes (S2 Fig). Second, we reasoned that if endogenous replication errors form unstable chromosomes preferentially at the T-403IR-C site, then randomized error along the chromosome should not generate such an enriched profile. Rather, random error would generate unstable chromosomes at random sites. To generate damage that may occur at many sites on a chromosome arm (and thus appear genetically to be random), we used methyl methanesulfonate (MMS) and hydroxyurea (HU) treatments. The errors by these drugs may be at specific sites, tRNA genes for example, of which there are 12 along Chr VII’s left arm, some in each genetic interval, thus giving the effect of random error. MMS- and HU-treatment and replication error does induce a 10-fold increase in unstable chromosomes, as expected (Fig 2C). We would thus have expected generation of random unstable chromosomes, and then random (or more random) relic recombinants. In fact we did find that allelic recombinant distributions (indicating sites of initial error) after MMS or HU exposure are generally more random than in untreated cells (with the exception of wild type + HU; S3 and S7 Figs). We do still detect an enrichment of allelic events at the chromosome end (perhaps telomeres are extra-sensitive to DNA damaging drug treatments, or telomere-repair events are more efficient than repair events along the arm; S3 and S7 Figs).

We then examined the relic recombinants from MMS and HU-treated sectored colonies. Surprisingly, drug-induced relic recombinants were still highly enriched in the T-403IR-C region (Fig 2A and S3 and S7 Figs). Why are relics recovered so often in the T-403IR-C region if MMS and HU induced damage occurs at many sites? There are two non-mutually exclusive explanations. It may be that the T-403IR-C site is hypersensitive to DNA damaging agents, as telomeres may be (mentioned above, although we did not see an MMS and HU induced increase in allelics in this interval). Alternatively, and a model we favor, events may initiate elsewhere and resolve preferentially in this internal region, which we now term a "collection site" (explained in the Discussion).

It is possible, therefore, that the T-403IR-C site undergoes spontaneous error to initiate events. If so, loss of the T-403IR-C site should abrogate unstable chromosome formation (as well as allelic recombinant formation). In contrast, if unstable chromosomes initiate elsewhere and frequently resolve in the T-403IR-C region, deletion of the T-403IR-C site would not affect the frequency of unstable chromosomes. We simply deleted the 4 Kb site from both Chr VII homologs, and tested instability in a variety of wild type and mutant cells (*rad9Δ*, *rad17Δ*, *rad18Δ*, and *rad51Δ*). The frequency of unstable chromosomes is decreased, at most 2-fold, in the mutants, and the proportion of allelic recombinants in the 94 Kb T-403IR-C region is significantly decreased (by 16% and 43% for the two T-403IR-C deletions tested); we conclude that the T-403IR-C site may be involved in initiation of less than half of allelic events (Fig 2C and S4 Fig). In addition, and remarkably, the enrichment of relic recombinants in the T-403IR-C region persists even in the absence of the 4 Kb T-403IR-C site (S4 Fig). We conclude that events likely initiate at many places on the chromosome arm (the T-403IR-C region being one hotspot), and frequently resolve in the T-403IR-C region.

### Telomerase defects induce unstable chromosomes

**Fig 3:**
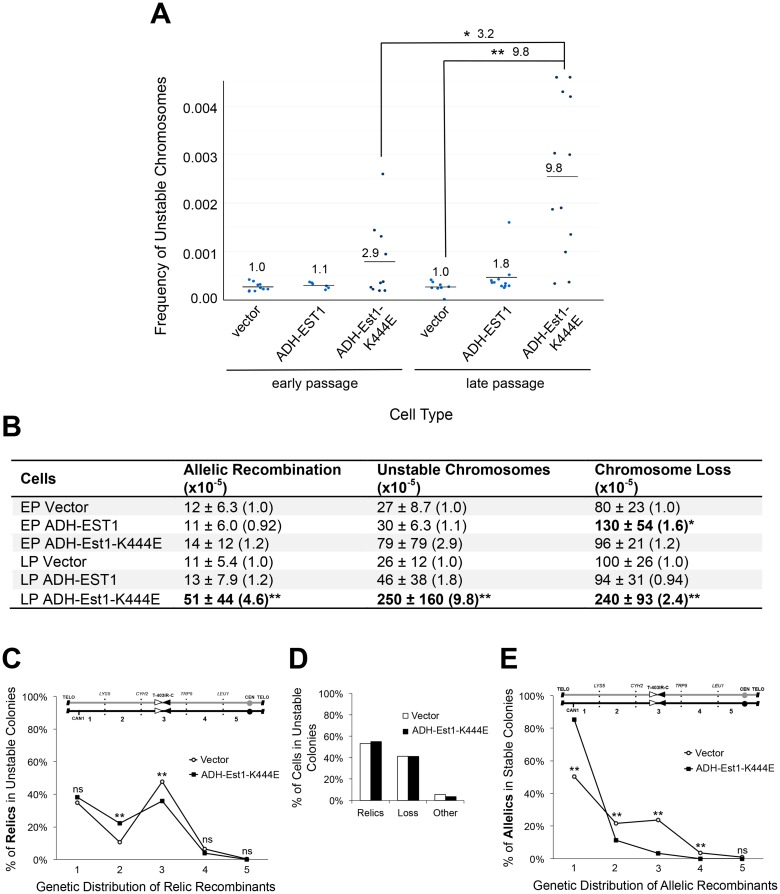
Telomerase defects cause chromosome instability. (A) The average frequency of unstable chromosomes (horizontal line) from wild type cells expressing telomerase alleles. Single points represent individual experiments. Wild type cells contain 2u plasmids of either vector alone (2μ HIS3); a wild type telomerase allele (ADH-EST1::HIS3); or a dominant negative allele (ADH-Est1-K444E::HIS3). Early and late passage cells grew ~70 and ~200 generations from transformation, respectively. Statistically significant differences are in brackets (*P < 0.05, **P < 0.01, Kruskal Wallis test). Fold changes between pairs of strains indicated above brackets, and within early or late passage group above mean bars. (B) Telomerase mutants induce all three forms of instability. Average frequency ± standard deviation is shown. Fold changes within respective passage group are shown in parentheses. Statistically significant differences are in bold (*P value < 0.05, **P value < 0.01, Kruskal Wallis test); EP: early passage cells, LP: late passage cells. (C) Genetic distributions of relic recombinants from LP vector (N = 291) and LP ADH-Est1-K444E (N = 373) Can^R^ Ade^+^ sectored colonies. Statistically significant differences between vector and ADH-Est1-K444E relic recombinants (and allelic recombinants in (E)) are shown above each genetic interval (**P < 0.01 or non-significant (ns), Z score test for population proportions). (D) Distributions of relic recombinants, loss or "other" recovered from Can^R^ Ade^+^ sectored colonies from vector (N = 549) and ADH-Est1-K444E (N = 645). (E) Genetic distributions of allelic recombinants from vector (N = 775) and ADH-Est1-K444E (N = 648) Can^R^ Ade^+^ round colonies.

Where might events begin? Given that errors and unstable chromosomes do not start in the T-403IR-C site, and the well-documented instability of telomeres, we reasoned that unstable chromosomes might initiate in the telomere. To test this idea, we disrupted telomere biology by introduction of a high copy plasmid overexpressing a telomerase dominant-negative mutant, in either of two telomerase subunits (ADH-Est1-K444E and ADH-Est3-R110A; [53,54]). Expression of the Est1 or Est3 mutant alleles in a wild type strain confers a short telomere phenotype, but does not induce senescence [53,54], bypassing this caveat of telomerase null strains (our assay requires > 60 cell divisions to identify and analyze unstable chromosomes). We found that telomerase mutants increased the frequency of unstable chromosomes by up to 10-fold compared to cells with high copy plasmid alone (vector), or high copy plasmid with a wild type telomerase subunit (either ADH-EST1 or ADH-EST3; Fig 3A and 3B and S5 Fig). We also found that the increase in unstable chromosomes occurs more prominently in late than in early passage cells (corresponding to shorter telomeres in cells from late passage compared to controls and early passage; S5 Fig). We conclude that the prolonged absence of telomerase renders something about the telomere, perhaps mere shortening, more prone to chromosome instability.

We next asked if unstable chromosomes in telomerase mutants resemble unstable chromosomes in wild type cells. To do so, we analyzed the distributions of relic recombinants from sectored colonies from telomerase mutant and wild type cells. We found relic distributions to be remarkably similar, enriched in the T-403IR-C region (Fig 3C and S5 Fig). We also note that allelic recombinants in wild type cells and in telomerase mutants are skewed toward the telomere (Fig 3E and S5 Fig), while the relic recombinants from both strains have an enhanced profile in the T-403IR-C region. The telomere enrichment of allelic recombinants is consistent with our suggestion that allelic recombinants arise at the initial site of error, in the telomere, while the relics indicate the structure of unstable chromosomes, some of which resolve to more stable forms in the T-403IR-C region. The common genetic distribution trends of allelic and relic recombinants in wild type and telomerase defective strains suggests that telomere defects may indeed be a common cause of unstable chromosomes. The identification of both unstable chromosomes and relic recombinants in telomerase mutants was not made in an earlier study ([27,28]; see Discussion and S1 Appendix).

### Telomere-proximal unstable chromosomes are common, even in telomerase^+^ cells, and are extremely unstable

**Fig 4:**
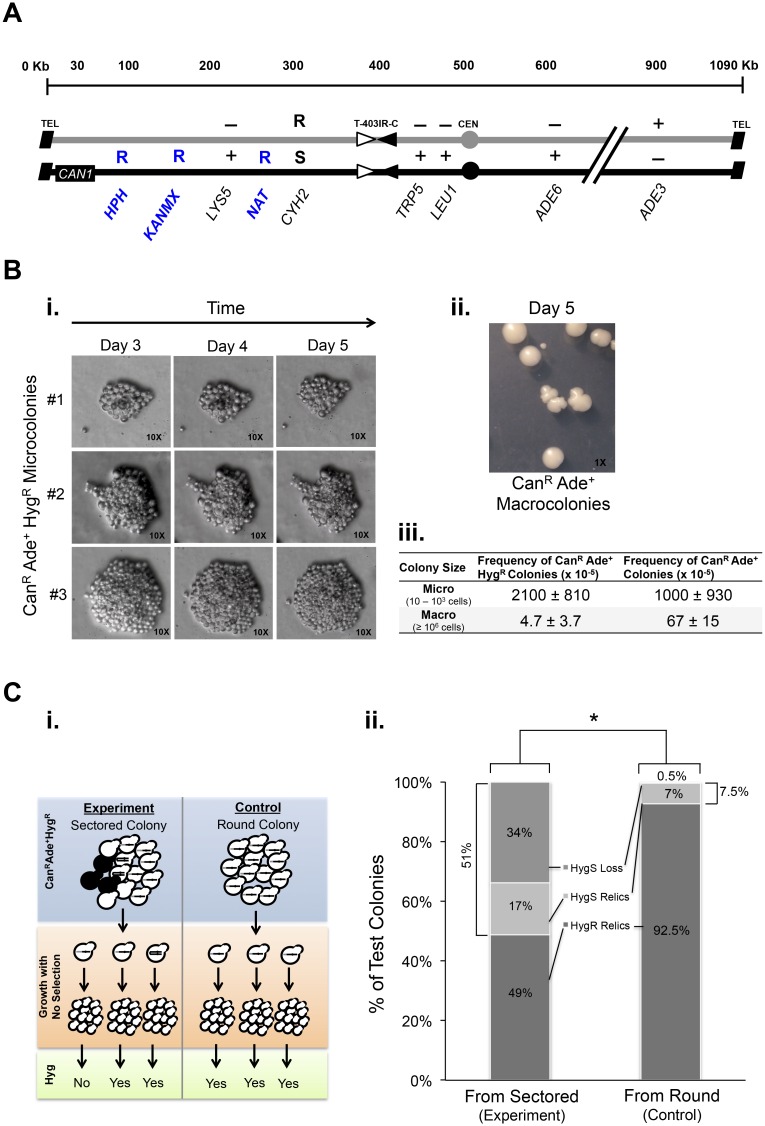
Longer Hyg^R^ unstable chromosomes are highly unstable. (A) Diagrams of Chr VII homologs engineered with additional genetic markers (Hyg^R^ (*HPH*) at 75 Kb, Geneticin^R^ (*KANMX*) at 120 Kb, and Nat^R^ (*NAT*) at 280 Kb). (B) i. Three representative microcolonies that ceased growing after day 3 on Can-Ade+Hyg selective plates. ii. Macrocolonies after day 5. iii. The average frequency ± standard deviation of micro and macro colonies from Can-Ade+Hyg selection and Can-Ade selection. (C) Protocol and results of test of instability of Hyg^R^ unstable chromosomes. i. Cells from sectored (experimental) or round (control) colonies (top) were plated onto rich media and allowed to form colonies (middle) that were subsequently phenotyped by replica plating (bottom). In this hypothetical example, 2 of 3 and 3 of 3 experimental and control cells, respectively, formed Hyg^R^ colonies ii. Bar graph shows the average percent of Hyg phenotypes from rich-media grown colonies derived from sectored colonies (N = 1537) or round colonies (N = 2195). Four independent experiments were performed. Statistically significant difference *P < 0.01 of Hyg^R^ colonies between stable and unstable populations, Z test for differences between two proportions.

To address the possible telomere-initiation of events, we sought to identify the earliest possible unstable chromosomes, and determine where they arise. We inserted markers in *rad9Δ* mutants, including the Hyg^R^ gene, *HPH*, 77 Kb from the telomere, to allow selection for telomere-proximal Hyg^R^ unstable chromosomes (Fig 4A). We grew *rad9Δ* cells in rich media and allowed them to form unstable chromosomes as before. Then we selected for loss of *CAN1* but retention of *HPH* by growth on selective solid media supplemented with hygromycin (Can-Ade+Hyg selection, as well as selection for *KanMX*, Geneticin^R^, and *NAT*, Nat^R^).

We examined Can^R^ Ade^+^ Hyg^R^ cells in sectored colonies for evidence of telomere-proximal unstable chromosomes. We made two observations consistent with a telomere-proximal unstable chromosome. First, we examined by light microscopy cells and colonies that must contain Can^R^ Ade^+^ Hyg^R^ chromosomes. Surprisingly, we found that about 2% of *rad9Δ* cells form Can^R^ Ade^+^ Hyg^R^ microcolonies (with between 10 and 10^4^ cells) on the Can-Ade+Hyg solid media (Fig 4B). The initial Can^R^ Ade^+^ Hyg^R^ cell either forms a microcolony or a macrocolony. If the Can^R^ Ade^+^ Hyg^R^ cell loses Hyg^R^ while growing on selection plates, the cell generates a microcolony. If the Can^R^ Ade^+^ Hyg^R^ cell retains Hyg^R^ while growing on selection plates, the cell generates a macrocolony. Thus, we suggest that Hyg^R^ unstable chromosomes form frequently, progress though multiple cell divisions, and then cease division due to loss of the Hyg^R^ gene (and/or Geneticin^R^ and Nat^R^ genes), forming microcolonies. The high frequency of microcolonies in *rad9Δ* mutants suggests many events (~1 in 50 cells) initiate in the last 77 Kb of the chromosome.

From the high frequency of Can^R^ Ade^+^ Hyg^R^ aborted microcolonies we suggest that Hyg^R^ unstable chromosomes are rapidly lost, causing the abortion of colony growth. We cannot directly test the fate of Hyg^R^ unstable chromosomes in those microcolonies. Yet, we can test the fate of Hyg^R^ unstable chromosomes if they persist in some macrocolonies (sectored colonies). We recovered cells from a Can^R^ Ade^+^ Hyg^R^ sectored colony that must contain cells with the Hyg^R^ gene. To test if the Hyg^R^ gene is on an unstable chromosome, we allowed cells to divide on rich media (no selection); if the Hyg^R^ gene is unstable, it may be lost during these rich-media cell divisions. Remarkably, we found that most Can^R^ Ade^+^ Hyg^R^ cells lost the Hyg^R^ gene upon cell division; fully 51% of the cells from an initially Can^R^ Ade^+^ Hyg^R^ sectored colony, when grown in rich media, were now Hyg^S^ (Fig 4C). The Hyg^R^ gene is not inherently prone to loss, as a cell from a Hyg^R^ allelic recombinant colony retains the Hyg^R^ gene when grown in rich media (Fig 4C). We conclude, therefore, that Hyg^R^ unstable chromosomes form frequently (~2% of *rad9Δ* cells) and are extremely unstable. Collectively, these data suggest that, like events in telomerase and *tel1Δ* mutants, events in *rad9Δ* mutants commonly initiate in or near the telomere (though events may initiate internally as well), and the initial unstable chromosome is extremely unstable.

### Extreme instability in *tel1Δ rrm3Δ* double mutants

**Fig 5:**
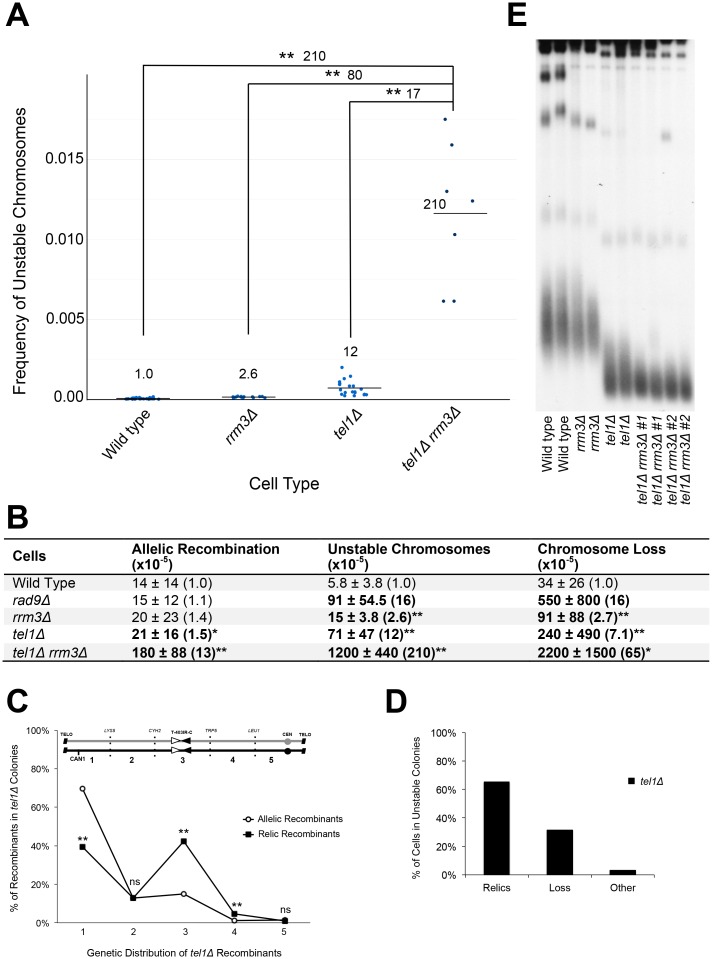
Synergistic increase in unstable chromosomes in *tel1Δ rrm3Δ* double mutants. (A) The average frequency of unstable chromosomes (horizontal line) from wild type and mutant cells. Single points represent individual experiments. Statistically significant differences are in brackets (**P < 0.01, Kruskal Wallis test). Fold changes shown above brackets. Fold changes between wild type and mutant strains are shown above mean bars. (B) Frequency of three instability events in *tel1Δ rrm3Δ* mutants. Average frequency ± standard deviation shown. Fold changes are in parentheses and are relative to wild type. Statistically significant differences are in bold (*P value < 0.05, **P value < 0.01, Kruskal Wallis test). (C) Genetic distributions of *tel1Δ* allelic recombinants (N = 1045) and relic recombinants (N = 416) in specific genetic intervals from Can^R^ Ade^+^ round and sectored colonies, respectively. Statistically significant differences between allelic and relic recombinants are shown above each genetic interval (**P < 0.01 or non-significant (ns), Z score test for population proportions). (D) Distributions of relic recombinants, loss, or “other” recovered from *tel1Δ* Can^R^ Ade^+^ sectored colonies (N = 638). (E) Telomere length analysis. Southern blot analysis of XhoI-digested DNA from exponentially growing cells, using a poly (GT) telomere-specific probe. Two independent colonies of each strain were analyzed. Two strains of *tel1Δ rrm3Δ* were analyzed.

To further test the link between DNA replication error and telomeres, we turned to the study of Tel1 and the DNA helicase, Rrm3. Tel1 is a protein kinase that regulates telomere length as well as the cell’s response to DNA double-strand breaks and terminal replication forks [15,55–58]. *Tel1Δ* mutants have a similar increase in unstable chromosomes as *rad9Δ* mutants ([59] and Fig 5A and 5B); an initially surprising result because, unlike *rad9Δ* mutants, *tel1Δ* mutants do not have a global repair defect, and we thought events initiated internally. Rrm3 is a DNA helicase that prevents fork stalling at many sites in the genome, including at telomeres [10,60,61]. We previously reported a modest increase in unstable chromosomes in *rrm3Δ* single mutants, consistent with a role for replication error and fork stalling in forming unstable chromosomes ([52] and Fig 5A and 5B).

We first characterized the allelic and relic recombinants formed in *tel1Δ* mutants. We found that *tel1Δ* mutants have an extremely high frequency of allelic recombinants enriched in the most telomere-proximal interval, consistent with a prominent role for Tel1 in preventing instability by acting at the telomere (Fig 5C and S7 Fig). And, similar to other mutants and random DNA error, *tel1Δ* sectored colonies generate relic recombinants with a substantial enrichment in the T-403IR-C region (Fig 5C).

To test if there is a link between Tel1 and Rrm3 in preventing unstable chromosomes, we generated a *tel1Δ rrm3Δ* mutant. We found a dramatic synergy of unstable chromosome formation; the double mutants are at least 20-fold more unstable than either single mutant (Fig 5A and 5B). There are two possible explanations for the high frequency of unstable chromosomes in *tel1Δ rrm3Δ* double mutants. First, Rrm3 may be needed to prevent replication error at telomeres in *tel1Δ* mutants. Alternatively, Tel1 may be needed to minimize instability at any of the many sites where Rrm3 inhibits fork stalling. To test between the two explanations, we asked if *tel1Δ rrm3Δ* double mutants showed an exaggerated telomere defect relative to either single mutant. We found that telomeres of *tel1Δ rrm3Δ* double mutants are as short as *tel1Δ* mutants (Fig 5E); we do not know if they are shorter. Another test if *tel1Δ rrm3Δ* instability arises in the telomere would be from allelic recombinant profiles. Unfortunately, *tel1Δ rrm3Δ* are so unstable we cannot analyze them for technical reasons (S7 Fig). We favor the view that events in the *tel1Δ rrm3Δ* initiate in the telomere, and await confirmation from other approaches. We also tested if an *rrm3Δ* mutation synergizes with the ADH-Est3-R110A allele, and did not detect synergy (S7 Fig), suggesting Tel1 and Rrm3 have an interaction distinct from a telomerase defect and Rrm3. In sum, for both *rad9Δ* and *tel1Δ rrm3Δ*, evidence suggests telomere errors form unstable chromosomes, yet its interpretation remains ambiguous as errors in these mutants might also arise elsewhere on the arm to form unstable chromosomes.

### Telomere-proximal unstable chromosomes progress to centromere-proximal unstable chromosomes and resolve to relic chromosomes

Finally, we wished to test if telomere-proximal unstable chromosomes progress to shorter unstable chromosomes (Fig 1B). We have presented some evidence of progression; telomerase-defective induced sectored colonies contain relic recombinants in the T-403IR-C region (Fig 3C and S5 Fig). Here we test by additional methods if a longer unstable chromosome can convert to the shorter, specific T-403IR-C dicentric chromosome [35].

**Fig 6:**
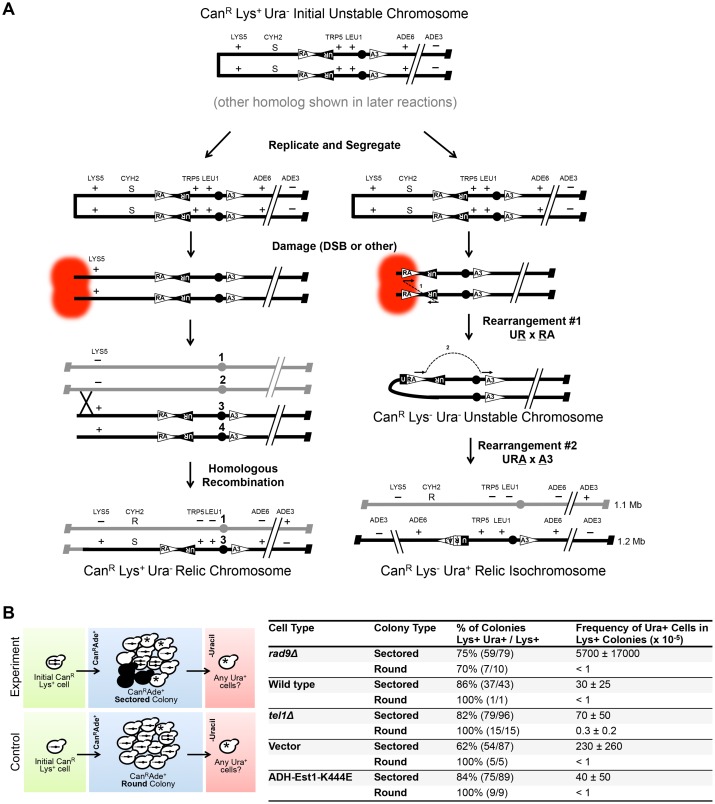
Longer unstable chromosomes progress to a shorter unstable dicentric chromosome. (A) Model of the generation of Lys^+^ Ura^-^ and Lys^-^ Ura^+^ relic recombinants from a single long unstable chromosome. The *URA3* gene was divided into three fragments and inserted into the LTR regions of Chr VII as discussed in the text and [35]. An initial (or early) fusion between Chr VII sisters of the *CAN1* homolog forms a Can^R^ Lys^+^ Ura^-^ unstable chromosome and is replicated. During mitosis, one unstable chromosome is segregated into each cell. Left: A break or lesion forms near the chromosome end and the lesion is repaired by recombination with the intact homolog to generate a Can^R^ Lys^+^ Ura^-^ relic recombinant. Right: A break or lesion forms near the UR and RA gene fragments and is aberrantly repaired, forming a fusion between the fragments of sister chromatids (Rearrangement #1) that generates a shorter unstable dicentric. The Lys^-^ URA unstable dicentric then undergoes Rearrangement #2 to form the Can^R^ Lys^-^ Ura^+^ relic isochromosome. See S8 Fig for further details of the conversion of a long unstable chromosome to a shorter unstable dicentric. (B) Scheme to test what fraction of sectored and round colonies had Ura^+^ and Lys^+^ cells. Table shows the percent of sectored colonies that had both Lys^+^ and Ura^+^ cells, divided by total Lys^+^ cells, and how many Ura^+^ cells per colony. Average frequency ± standard deviation of Ura^+^ cells from Can^R^ Ade^+^ sectored colonies that contained any Lys^+^ cells.

To demonstrate that the single long unstable chromosome can progress and form the shorter specific dicentric, we use the logic and genetic constructs shown in Fig 6A (and S8 Fig in slightly more detail). We use a telomere-proximal marker, *LYS5*, 220 Kb from the telomere; detection of Lys^+^ relics indicates there once was a telomere-proximal unstable chromosome. We use a previously described set of DNA fragments that ultimately form a *URA3* gene if a dicentric forms in the T-403IR-C site; detection of Ura^+^ cells indicates there once was a centromere-proximal unstable chromosome. Referring to Fig 6A, we surmise that, if both Lys^+^ Ura^-^ and Lys^-^ Ura^+^ cells come from one sectored colony, then a longer unstable chromosome was formed, duplicated, and each duplicate had a separate fate, as shown.

To summarize the *URA3* system briefly: we found previously that in sectored colonies, some cells undergo a recombination event between two inverted LTRs in the T-403IR-C site to form a dicentric, followed by recombination between two direct-repeat LTRs to delete one centromere and form an isochromosome (S8 Fig, [35,52]). Fragments of *URA3* were constructed to mimic the LTR recombination events, and we showed they do ([35] and Fig 6A and S8 Fig).

To determine if a common unstable chromosome forms both Lys^+^ and Ura^+^ relics, we simply screened *rad9Δ* Can^R^ Ade^+^ sectored colonies for both (Fig 6B). We found, in fact, that most sectored colonies contained some Lys^+^ and Ura^+^ cells. (Ura^+^ cells were verified to contain a 1.2 Mb Ura^+^ translocation; Fig 6B and S8 Fig). (Importantly, the frequency of Ura^+^ cells in the sectored colonies was about 5%, whereas the frequency of Ura^+^ cells in round colonies was less than 0.001% (~10^4^ lower)). The Ura^+^ cells in round colonies, performed as a control, probably arise infrequently after an unstable chromosome forms from the stable Can^R^ Ade^+^ chromosome. And as expected, Can^S^ Ura^-^ cells also very infrequently convert to Ura^+^ (less than 1 in 10^5^ cells [35]). We conclude that a single unstable chromosome can generate both Lys^+^ relics arising from long unstable chromosomes, as well as the Ura^+^ isochromosome relic arising from a shorter unstable dicentric chromosome.

Using this Lys^+^ and Ura^+^ criteria, we next analyzed telomerase mutants, *tel1Δ* mutants, and wild type cells for progression of longer unstable chromosomes to shorter dicentrics to the Ura^+^ isochromosome. In each strain we again identified sectored colonies that had both Lys^+^ and Ura^+^ cells. And, we again found a higher frequency of Ura^+^ cells from sectored colonies than from round colonies, suggesting that longer unstable chromosomes frequently convert to the shorter dicentric. And, the Ura^+^ cells had a 1.2 Mb isochromosome, confirming the dicentric to isochromosome event (Fig 6B and S8 Fig). We did find that the frequencies of Ura^+^ cells in sectored colonies in wild type, *tel1Δ* and telomerase mutant strains are not as high as in *rad9Δ* mutants, for unknown reasons (S8 Fig). Nevertheless, we conclude that longer unstable chromosomes, most of which probably initiate in the telomere, generate both longer relics (Lys^+^) and shorter, unstable dicentric chromosomes resolved to the isochromosome (Ura^+^).

## Discussion

**Fig 7:**
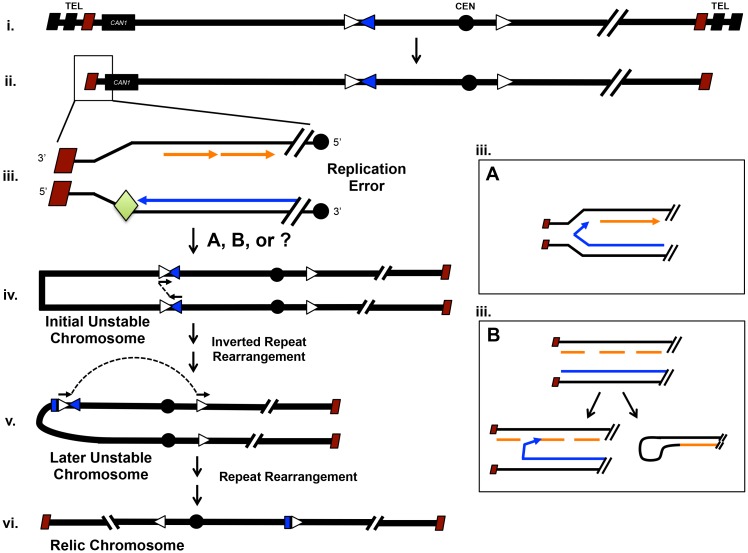
Telomere replication error generates unstable chromosomes and global instability. In i to ii: as telomeres shorten (3 rectangles reduced to 1) unresolved replication errors arise at the chromosome end (iii; green diamond represents some error). The, replication errors may arise at the fork (iiiA) or after the fork (iiiB). This forms an initial unstable chromosome (iv) that undergoes further instability. The unstable chromosome has multiple possible fates (see Fig 1B); the one shown here forms the isochromosome (iv to v to vi). Symbols: Terminal TG repeats of telomere: black diagonal rectangles; Internal-most TG repeats of telomere: maroon diagonal rectangles; Obstacle to the replisome: green diamond; Lagging strand synthesis: orange arrows; Leading strand synthesis: blue arrow; T-403IR-C inverted repeats (Ty3 LTR sequences, Fig 1A): open and blue arrowheads.

In this study we provide evidence that replication errors in or near the telomere generates unstable chromosomes, easily detected in a Chr VII disome system. The initial unstable chromosomes are either lost, resolve by recombination to form relic recombinants, or progress to shorter unstable chromosomes that, in turn, may resolve in a centromere-linked T-403IR-C region we term a "collection site" (Fig 7). The evidence for this model is that first, unstable chromosome formation is increased in telomerase and *tel1Δ* mutants (Figs 3 and 5 and S7 Fig), both with telomere-prominent roles (Figs 3E and 5C and S5 Fig). Even *rad9Δ* mutants with no telomere-specific function may often initiate unstable chromosomes near telomeres (Fig 4). Second, a *tel1Δ* mutation synergizes with an *rrm3Δ* mutation to form extremely high frequencies of unstable chromosomes, furthering the link between DNA replication and telomere errors (Fig 5A and 5B). Finally, initial longer unstable chromosomes can convert to shorter unstable chromosomes (Fig 6).

We also clarify the role of the T-403IR-C site and region as what we call a collection region, where events that begin elsewhere resolve to stable chromosomes. Even random errors generated by MMS and HU form unstable chromosomes that resolve in the collection region. We speculate here on the nature of replication error in telomeres that leads to unstable chromosomes, and how long unstable chromosomes might progress to short unstable chromosomes. (Also see S4 Fig for comments on the T-403IR-C region being not only a site of initiation but also of resolution, or collection, of unstable chromosomes that initiate elsewhere.)

### Telomere error is a common origin of unstable chromosomes in budding yeast

We propose that telomere error, as opposed to random error along the arm, is perhaps the most common origin of unstable chromosomes in budding yeast. Telomere sequences in cells mutant for telomere maintenance proteins are known to be unstable; they fuse in budding yeast [29,32] and in mammalian cells [26,33], and form unstable chromosomes (this study). Not unexpectedly, telomeres are a major source of rearrangements in evolution [62,63] and experimentally [64,65]. And we note that for telomere error in telomerase mutants, ~80% of the recombinants are unstable chromosomes, and ~20% are allelic recombinants (Fig 3 and S5 Fig). We infer all together that telomere error is a predominant source of instability in the genome, in particular in the formation of unstable chromosomes. That telomeres are the major source of instability is not testable, as there is not a method to test instability along every kilobase of a chromosome, to our knowledge.

Why are telomeres such a common source of error? We, and others, posit two explanations, discussed in turn below. First, telomerase itself may prevent replication error, or facilitate repair of replication error, by somehow interacting with the replisome and the telomere. Second, shorter telomeres per se may simply leave the chromosome end susceptible to degradation.

### Telomere replication error and instability

Our previous extensive genetic analysis suggested that instability initiates following DNA replication errors [35,36,52]. Our current reevaluation of unstable chromosomes connects replication error with the telomere. There is of course ample precedence for problematic replication through telomeric sequences; the loss of telomere binding proteins and replication factors causes replication fork failure and thus telomere fragility in mammalian cells, in budding and in fission yeasts [10–14].

How might replication of telomeres generate instability, in particular unstable chromosomes? We imagine two models, one of events at a failing replication fork and the second of events behind the failed replication fork (Fig 7). Error at the fork may form a so-called "closed fork", caused by the misannealing of the 3’ end of the leading strand to the lagging strand template. Or error at the fork may form a reversed fork first, caused by annealing of the newly synthesized leading and lagging strands, and then form a closed fork (Fig 7 Box A) [15,49]. Reversed forks are popular in many models of fork error [4]. Alternatively, a failing fork may replicate to chromosomes' end, and leave error (e.g. single-stranded gaps or DNA double-strand breaks). The damaged chromosome may then fuse to form dicentrics by any of several imaginable mechanisms (a faulty template switch or fold-back hairpin in Fig 7 Box B; [35,47,51]). Our elusive unstable chromosome may be either a bonafide dicentric made as the fork fails, or may be a linear chromosome with gaps formed behind the failed fork, or some other unknown structure.

We cannot readily distinguish between the various models. For example, defects in the Rrm3 DNA helicase increases formation of unstable chromosomes, though it is not known if *rrm3Δ* mutants are more prone to forming a closed fork structure, or preferentially leave gaps in fully replicated chromosomes. Similar ambiguity in mechanistic interpretation accompanies mutants in Rad9, Mec1, Rad53, Rad18 and many other proteins required for maintenance of chromosome stability [35,36,52,59]. The only mechanistic clue we currently have is that unstable chromosomes typically form in some mutants independent of non-homologous end joining (NHEJ), homologous recombination (HR), and single strand annealing (SSA). Thus, we proposed a "faulty template switch" as in the closed fork model, to form dicentrics [35]. Mutants like *tel1Δ rrm3Δ* with extremely high frequencies of instability potentially provide tools to identify unstable chromosome and further define what happens to failed replication in telomeres.

### Might telomere-initiated instability be caused by genome-wide replication stress?

We consider the possibility that telomere error causing unstable chromosomes may be indirectly induced by DNA replication stress in the whole genome. It was recently suggested that telomerase might move with the replication fork through the telomere [69]. It is possible that telomerase might even localize to an internal replication fork when there are errors in TG rich sequences. This might result in the titration of telomerase away from the telomeres towards internal sites. The competition for telomerase could lead to telomere shortening and then terminal replication error [15]. Is there evidence for genome-wide replication stress causing telomere instability? We find it curious that cells treated with MMS and HU still suffer an enrichment of telomeric allelic recombinants (in addition to relics enriched at the collection site; S3 and S7 Figs). Characterization of the relationship between telomere maintenance and genome-wide replication stress awaits further study.

### To what extent is instability due to telomere resection or to unstable chromosomes?

Telomere dysfunction does result in chromosome end degradation [19,21]. A previous study related directly to our current study concluded, in fact, that telomerase defects in budding yeast cause chromosome instability by permitting end degradation. Hackett, Feldser, and Greider carried out elegant experiments that led to this conclusion [27,28]. They even generated a dicentric artificially, showing that it generates recombinants all along the chromosome arm, while a telomerase defect generated recombinants only near the telomere. They provided genetic evidence that instability arose due to end degradation (reduced in an *exo1Δ* mutant), and not dicentric formation [28].

The results of our study suggest a different conclusion, that a telomerase defect generates two consequences. First, a telomerase defect causes allelic recombination near the telomere, as seen by Hackett, et al. [27,28]. And, a second consequence not detected by Hackett, et al., a telomerase defect causes formation of unstable chromosomes, and dicentrics, leading to chromosome-wide changes. The role of degradation per se we address below. Why do our two studies differ in conclusions? There are a myriad of technical differences we think minor (different telomerase alleles; diploids versus disomes; though the same Chr VII arm was used in both studies). Yet the key major difference we believe is the following: we can detect both stable (allelic) and unstable recombinants (generating relics), while they detected only stable allelic recombinants; the generation and fate of unstable chromosomes was completely missed in their study (S1 Appendix). Unstable chromosomes account for about 80% of the rearranged chromosome products recovered from a telomerase defect (Fig 3B). Why can we detect unstable chromosomes but they could not? Our system generates a unique product, a slow growing but distinctive sectored colony, arising from unstable chromosomes that would be over-grown were it in the presence of allelic recombinants. Their system could not achieve separation of stable alleles from unstable chromosomes. Note that we do find, as they did find, that telomerase mutant cells generate mostly telomere proximal allelic recombinants (Fig 3E and S5 Fig).

### A speculative role for degradation in early steps of instability

It may be that telomere resection is involved in forming an early Can^R^ unstable chromosome. This supposition comes from a puzzling feature of the Chr VII disome and *CAN1*: given that the telomere and *CAN1* are ~30 Kb apart, how does telomere instability inactivate *CAN1*? There are of course many possible explanations, but little data to distinguish between them. DNA sequences near the telomere may interact with sequences centromere-proximal to *CAN1*, deleting the terminal > 30 Kb as an initial event. Such an interaction may involve looping of DNA, or DNA degradation. Or a first unstable chromosome may occur near the telomere, leaving *CAN1* intact, and then a second rearrangement eliminates *CAN1*. Studies where we can detect instability arising in a single cell cycle, using mutants with a high frequency of instability, may address the nature of the initial event(s) arising from the telomere and effecting *CAN1*.

### Unstable chromosome progression to a common collection region

We have shown that unstable chromosomes formed by telomere error progress ~400 Kb to an internal region of the chromosome. This progression may arise by either successive breakage-fusion-bridge cycles or extensive degradation. The traditional mechanism of progression is the breakage-fusion-bridge (BFB) cycle between dicentrics, first proposed by McClintock [22,42]. In the BFB model, an initial dicentric chromosome breaks and then the broken sisters fuse to form a second, shorter dicentric. The BFB cycle generates signature rearrangements, Kb long duplicated and then inverted repeats, that we have not detected in studies of Chr VII. The only abnormal structure we have recovered is the isochromosome that does not have extensive BFB-like repeats [52]. We have ruled out the canonical mechanisms of fusion (NHEJ, HR and SSA [35]) for at least some unstable chromosomes, though fusion between sister chromosomes might arise by some other mechanism (template switch of replication forks nearing a double-strand break, for example).

If progression does not arise by cycles of BFB, then how might a long unstable chromosome convert to a smaller one? First, progression might arise simply by degradation from the telomere. After degradation, the sisters might then fuse (perhaps by a template switch). The rate of degradation is believed to be about 4 Kb per hour; therefore conversion of a linear chromosome to a centromeric collection site 400 Kb away seems unlikely (requiring about 50 generation times of degradation). We find telomere-proximal unstable chromosomes convert to relics in the collection region in less than 20 generations. In addition, unstable chromosomes that are simply being degraded should activate the *RAD9* checkpoint and delay colony formation; we have not detected differences in sectored colony formation between *rad9Δ* mutants and other mutants with the checkpoint intact (e.g. *rad51Δ*). Thus, degradation per se from linear chromosomes seems an unlikely mechanism of progression. One prediction of a linear degradation model is that relic recombinants of different types would arise sequentially; we have not yet detected any such pattern, comparing relics from early and late sectored colonies.

It seems more likely that an unstable, perhaps dicentric, chromosome forms near the telomere, which then breaks more centromere-proximal. Degradation may ensue from the broken chromosome, enabling recombination in the collection region (allelic recombination or dicentric formation).

### The collection region

The enrichment of relics from unstable chromosome to the T-403IR-C region remains perplexing (Fig 2A and S2 Fig). The 4 Kb T-403IR-C site is clearly not the reason for enrichment, as it can be deleted with a modest effect on the frequency of instability and no effect on relic enrichment (Fig 2C and S4 Fig). A recent study mapping regions of dicentric breaks provides an hypothesis for the mechanism of unstable chromosome collection [41]. When a dicentric forms, it stretches between mother and daughter cell, and there may be nucleases at the bud neck that break the dicentric. The preferential collection site may thus be a consequence of dicentric size dictating its geometry to the bud neck, the site of nuclease cleavage, and the relics recovered.

### Ontogeny of unstable chromosomes reveals novel trajectories of instability

In conclusion, the Chr VII disome provides a model to study how replication fork error in telomeres leads to unstable chromosomes, and how initial unstable chromosomes progress. An additional unexplored implication of this work is the likely relationship of telomere defects causing instability to ageing; it is known that even in yeast older cells have greater instability [66]. Development of yeast strains that form unstable chromosomes in a single cell cycle, and at a high frequency (~1 in 30 cells), will provide methods to address the many remaining questions of replication, telomere and unstable chromosome biology, each of particular importance to ageing cells.

## Methods

### Yeast strains

Strains are derived from the A364a strain described previously [35,52,67]. The TY200 wild type Chr VII disome strain is *MATα +/hxk2*::*CAN1 lys5/+ cyh*^*r*^*/CYH*^*S*^ *trp5/+ leu1/+ cenVII ade6/+ +/ade3*, *ura3-52*. *CAN1* on Chr V has been mutated and inserted in one Chr VII homolog [68]. TY206 contains a *rad9Δ*::*ura3* null mutation generated from the TY200 starting strain. Additional strains were generated by LiAC/ssDNA/PEG transformation of TY200 or TY206 strains with DNA fragments or with plasmids. Strains were verified by genetic analysis, Southern analysis, and/or PCR. For all mutants reported, at least two separate strains were made and analyzed for similar phenotypes.

Telomerase defective strains were made by transformation of TY200 cells with high-copy 2μ plasmids (see S2 Table).

The extensively marked Chr VII disome (Fig 4A) was constructed by transforming *rad9Δ* cells with DNA fragments containing selectable markers flanked by 45 bp of homology to the targeted Chr VII locus [69]. DNA fragments were synthesized by PCR amplification of drug resistant genes from plasmids (*HPH* from pAG32, *KANMX4* from pRS400, *NAT1* from pmrc1NAT1) with primers containing 45 bp of homology to DNA of each targeted region. The hygromycin resistance gene (*HPH*) replaced Chr VII 75000 bp– 76050 bp, geneticin resistance gene (*KANMX4*) replaced Chr VII 121500 bp– 122100 bp, nourseothricin resistance gene (NAT) replaced Chr VII 286400 bp—287300 bp. Cells were then transformed and candidate drug resistant clones were verified by PCR using primers outside of the region. Genetic analysis was performed to verify selectable markers were integrated along the *CAN1* homolog (canavanine resistant colonies became sensitive to the selectable drugs: hygromycin, geneticin, and nourseothricin).

The *URA*3 inverted repeat module (Fig 6A and S8A Fig) was constructed as previously described (S5 Fig from [35]). Briefly, *URA3* gene fragments were joined to drug resistance genes to generate two cassettes (RA:Nat1:RU or A3:KanMX4) that were inserted into plasmids containing ~500 bp of sequence flanking sites 403 Kb or 535 Kb along Chr VII (RA-NAT1-RU within pRS406-403 and A3-KanMX4 within pRS406-535). Plasmids were digested with restriction enzymes to liberate the targeting fragment and TY200 cells were transformed and selected for drug resistance. Insertions into candidate drug resistant clones were verified by PCR and genetic analyses.

*tel1Δ* and *tel1Δ rrm3Δ* strains were generated by PCR amplification of KanMX4-marked gene replacements from the Euroscarf strain using primers that flank each gene. A *URA3* allele was introduced to replace the KanMX4 allele of the *tel1Δ* single mutant.

The inverted repeat deletion strains were generated by transformation of cells with DNA constructs containing drug resistance genes flanked by 45 bp of homology to the Chr VII region to be disrupted [69]. The *T-403IR-CΔ* spans Chr VII sequences 401498 bp to 405567 bp. The *T-320IR-CΔ* spans Chr VII sequences 318631 bp to 319390 bp. Inverted repeat regions were removed from both Chr VII homologs by consecutive transformations followed by genetic analysis to ensure selective markers integrated into the targeted Chr VII homolog and that all Chr VII disome auxotrophic markers were retained. Further, PCR was performed flanking the inverted repeat deletion regions to verify that selective markers replaced the regions. Karyotypes of strains were unaltered, as determined by Pulse Field Gel Electrophoresis.

The following drug concentrations were used for drug resistant selection of transformed cells: canavanine (Can; 60μg/mL), G418/geneticin (100μg/mL), hygromycin B (300μg/mL), and nourseothricin (Nat; 50μg/mL).

### Chromosome instability assays

Genetic analyses to determine the frequencies of unstable chromosomes, allelic recombinants, and chromosome loss were performed as described previously [52]. Briefly, single cells retaining both intact Chr VII homologs were plated to solid rich media (YEPD, 2% dextrose) and grown for 2–3 days at 30°C to form colonies. Chromosome changes occur as cells grow on solid rich media. Individual colonies were suspended in water, cells were counted with a haemocytometer and plated to media lacking arginine and serine to measure cell viability and selective media to measure instability. To measure cell viability, cells were grown overnight (~18 hrs) at 30°C. The average percentage of viable cells was determined by counting the number of microcolonies grown within a population (approximately 500 cells were observed per sample). To determine frequencies of chromosome loss, cells were plated to selective media containing canavanine (60μg/mL) and all essential amino acids except arginine and serine. Loss was determined following replica plating to genetically identify Ade^-^, Trp^-^, Leu^-^, Lys^-^ colonies. To determine frequencies of allelic recombination or unstable chromosomes, the selective media also lacked adenine. Cells were grown on selective media for 5 days at 30°C and then colonies were counted based on morphology (round or sectored, see Fig 1A). The frequency of chromosome events was calculated after normalizing the total number of cells plated by the percentage of cell viability. Average frequencies and standard deviations were determined from analysis of at least six colonies grown on solid rich media then plated to solid selective media. Statistical tests were performed using the Kruskal-Wallis method [70].

To determine frequencies of chromosome changes after exposure to HU or MMS, cells were plated to solid rich media with or without drug (YEPD, 2% dextrose; YEPD, 2% dextrose + 100mM Hydroxyurea; or YEPD, 2% dextrose + 0.01% Methyl methanesulfonate). Cells were incubated for 6 hrs at 30°C, and then washed from plates, rinsed with water twice, and plated to solid canavanine selective medias (with or without adenine) to determine frequency of chromosome events as described above.

To determine the frequencies of chromosome events in cells containing telomerase mutations, strains containing plasmids (Est1 and Est3 alleles and control vectors) were plated to solid media lacking either histidine (for strains containing the 2μ HIS3 vector, ADH-EST1::HIS3, or ADH-Est1-K444E::HIS3) or lacking uracil (for strains containing the 2μ URA3 vector, ADH-EST3::URA3, or ADH-Est3-R110A::URA3) to retain plasmids. Strains were passaged on each respective solid dropout media (-histidine or –uracil) for approximately 70 generations for early passage assays or 200 generations for late passage assays. Individual colonies were then plated to canavanine selective media to determine frequency of chromosome events as described above.

To determine frequencies of microcolonies on canavanine selective media plates, cells from the extensively marked *rad9*Δ cells (Fig 4A) were grown on rich media and individual colonies were then suspended in water, counted, and plated for viability and to selective media (as described above). About 10^5^ cells were plated to canavanine media lacking adenine, and ~8 x 10^5^ cells were plated to media containing canavanine, hygromycin, geneticin, and nourseothricin, but lacking lysine and adenine (fewer cells grow under the increased selection; S6 Fig). Cells were grown for 5 days at 30°C and were then assayed for macrocolony (sectored or round colonies) and microcolony growth. Macrocolony frequencies were calculated as described above. Microcolony frequencies were determined by counting the number of microcolonies within a microscopic field of vision (microcolonies were defined as cell clusters containing ≥ 10 cells and < 10^6^ cells and not visible macroscopically by eye.) At least 400 cell populations were observed per plate and then the average percentage of microcolonies per population was calculated. For example, if four fields of vision are observed with: 5/100 (5%), 4/150 (2.7%), 4/100 (4%), and 4/150 (2.7%) microcolonies then we estimate that an average of 3.6% (frequency of 3.6 x 10^−2^) of the cell population plated forms microcolonies. Average frequencies and standard deviations were determined from the analysis of at least six colonies.

### Phenotypic analysis of altered chromosomes

Pooled Lineage Analysis: Chromosome instability assays were performed from 6 or more individual rich-media grown colonies per strain. Then, cells from approximately 50 round or sectored Can^R^ Ade^+^ colonies were pooled, and suspended in water. Approximately 100 cells were then plated to each of four or five solid rich media plates, and grown for 2–3 days to form colonies. Thus, about 400–500 cells from round or sectored colonies were allowed to form colonies. Colonies were replica plated to synthetic media or media containing drugs (hygromycin, geneticin, or nourseothricin) and grown for 2 days at 30°C, and growth assessed. For example, a colony that was replica plated and growing on -lysine, -tryptophan, -leucine, and -adenine solid dropout media, but not growing on -adenine dropout media containing cychloximide drug would be scored as having a recombination event within the first interval of the Chr VII disome (this would result in loss of *CAN1* but retention of all other Chr VII disome genetic markers (Fig 2A), including *CYH2* allele which confers sensitivity to cycloheximide drug). The percentage of allelic recombinants or relic recombinants per pooled population was determined by dividing the number of genetic recombinants per interval by the total number of recombinant colonies assayed in the pooled population (the total number of recombinant colonies does not include colonies that had lost the chromosome or colonies with “other” genotypes). “Other” genotypes refer to complex genotypes in which multiple genotypes were found within a single colony (for example, a single colony had ~50% of cells with a relic recombinant genotype and ~50% of cells with chromosome loss). In these "other" colonies, the initial cell from the sectored colony must have been still unstable (a phenotype we called "fragmented" in [52]).

Z scores were calculated to determine the statistical significance between the proportion of Hyg^R^ populations from stable and unstable colony pooled lineages in Fig 4C and between allelic and relic recombinant distributions (Figs 2, 3 and 5 and S2–S5 and S7 Figs).

Genetic Marker Retention: To determine the presence of genetic markers within cells (as in Fig 6B and S6 Fig) in which even one cell retained a marker, entire individual round or sectored Can^R^ Ade^+^ colonies (~10^6^ cells/colony) were suspended in microtiter wells containing 50μl water and replica-pinned onto solid selective media (~10^5^ cells replica-pinned). Cells were grown for 2 days at 30°C and then scored for presence or loss of genetic marker based on growth of at least one colony on each selective media. To determine quantitatively the frequency of cells retaining genetic markers within round or sectored colonies (as in Fig 6B), cells from individual colonies were suspended in water, counted by haemocytomer, and then cells were plated to solid media selecting for each drug resistance or auxotrophic marker. Cells were grown for 2 days at 30°C and the number of colonies grown on selective media were counted.

### Imaging of arrested microcolonies

Approximately 8 x 10^5^ *rad9Δ* cells (with extensively marked Chr VII disome homologs, see Fig 4A) from individual colonies grown on rich media (YEPD, 2% dextrose) were suspended in water and plated to selective media containing canavanine, hygromycin, geneticin, and nurseothricin, but lacking lysine and adenine. Cells were grown for 3 days at 30°C and then observed for microcolonies (~1 cell– 1000 cells). Microcolonies were manually demarcated and imaged daily from 3d—5d of growth at 30°C.

### Pulsed field gel electrophoresis and southern blot analysis of altered chromosomes

To identify potentially altered chromosomes, cells from Can^R^ Ade^+^ sectored colonies (S4 Fig) or from Ura^+^ cells isolated from Can^R^ Ade^+^ sectored colonies (S8 Fig) were grown to stationary phase in 5 mLs of selective media lacking adenine. DNA agarose plugs were prepared and chromosomes were separated by pulsed field gel electrophoresis using conditions that optimize for separation of 1100 Kb (native) and 1200 Kb (translocation) chromosome sizes. Standard southern hybridization conditions were performed using a ^32^P-labeled probe to Chr VII sequences 503875 bp– 505092 bp.

### Southern blot analysis of telomere length

To determine the length of telomeres in Fig 5 and S5 Fig, genomic DNA was prepared from two individual colonies per strain (growth conditions are described for each strain below) and digested with XhoI restriction enzyme. XhoI-digested genomic DNA was subjected to 0.8% agarose gel electrophoresis and hybridized with a ^32^P-labeled poly(GT) probe. Standard hybridization conditions were used.

Fig 5 strain growth conditions: wild type, *rrm3Δ*, *tel1Δ*, or *tel1Δ rrm3Δ* cells were grown from freezer stock. Individual colonies were grown on solid rich media, isolated and then grown in liquid rich media from which genomic DNA was prepared. Each mutant strain had grown for approximately 80 generations after null deletion at the time of genomic DNA prep. S5 Fig strain growth conditions: wild type cells were transformed with either the 2μ HIS3 or 2μ URA3 vectors or the ADH-Est1-K444E::HIS3 or ADH-Est3-R110A::URA3 plasmids and were passaged for either ~70 generations (early passage) or ~200 generations (late passage). Individual colonies were grown on dropout medium (lacking either histidine or uracil), isolated, and then grown in liquid dropout media from which genomic DNA was prepared

## Supporting Information

S1 Fig The selection scheme used to study chromosome changes. (PDF)

S2 Fig Distinct ontogenies of allelic and relic recombinants. (PDF)

S3 Fig Recombinant genetic distributions in wild type and *rad9Δ* are unchanged after random damage. (PDF)

S4 Fig Unstable chromosomes form in the absence of T-403IR-C site with its tRNA genes and inverted repeats. (PDF)

S5 Fig Unstable chromosomes are induced by telomerase defects. (PDF)

S6 Fig Longer unstable chromosomes are highly unstable. (PDF)

S7 Fig Telomere dysfunction and replication defects induce unstable chromosomes. (PDF)

S8 Fig Longer unstable chromosomes resolve to an isochromosome. (PDF)

S1 Appendix Unstable chromosomes were missed in previous studies. (PDF)

S1 Table *Saccharomyces cerevisiae* strains used in this study. (PDF)

S2 Table Plasmids used in this study. (PDF)
