# Supplementary material for: Ontogeny of Unstable Chromosomes Generated by Telomere Error in Budding Yeast
Source: PLoS Genet. 2016 Oct 7;12(10):e1006345. doi: 10.1371/journal.pgen.1006345 (PMC5065131; doi:10.1371/journal.pgen.1006345)
Supplement: S1 Fig — (PDF) [file pgen.1006345.s001.pdf]

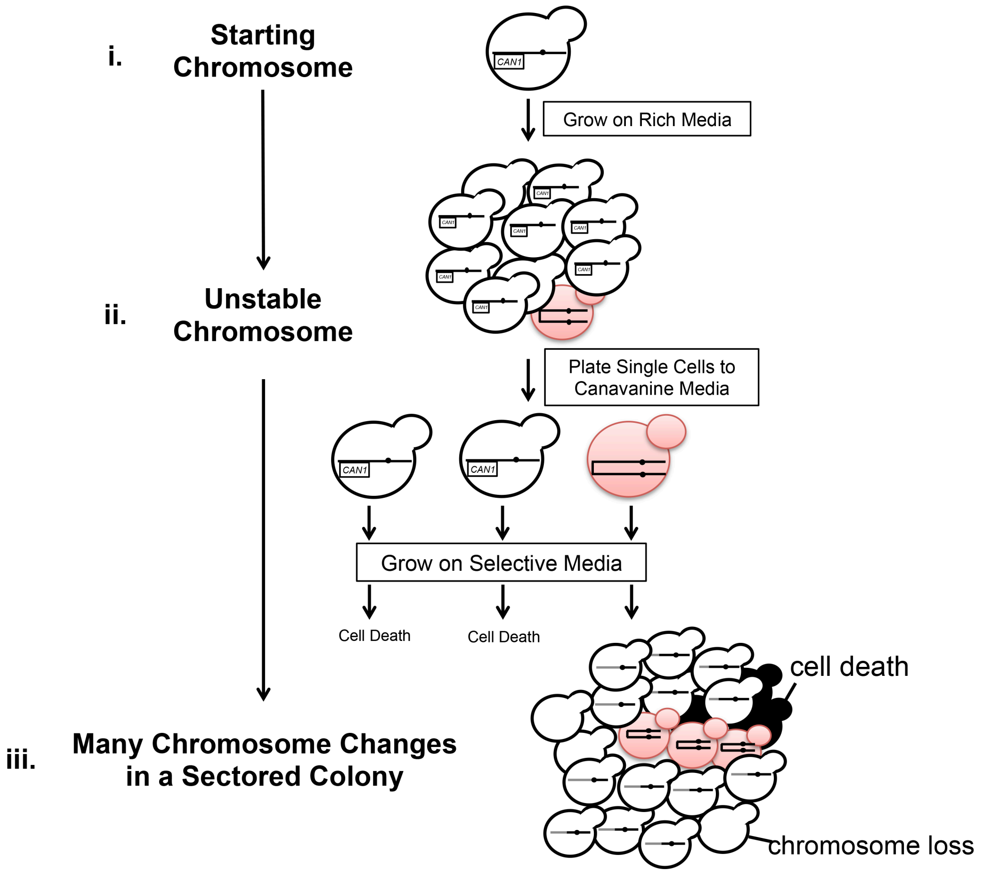

S1 Fig. The selection scheme used to study chromosome changes. i. Cells starting with both Chr VII homologs (only the *CAN1* homolog is shown) are grown on non-selective rich media. As cells divide to form a colony, spontaneous chromosome changes arise. The red cell in the colony contains an unstable chromosome (shown as a dicentric). **ii.** Cells are then plated to canavanine media to select for chromosomes that have lost *CAN1*. Shown are three cells; the first two cells die on the selective media because they retained *CAN1* (Can^S), but the red cell had lost *CAN1*, survives, and contains the unstable chromosome. **iii.** As that red cell then divides on selective media, the unstable chromosome incurs further rearrangements (see the five fates of unstable chromosomes, Figure 1B). Thus, the sectored colony formed contains cells with many different chromosome rearrangements, as well as cells that have lost the chromosome or have died.
