# Supplementary material for: Ontogeny of Unstable Chromosomes Generated by Telomere Error in Budding Yeast
Source: PLoS Genet. 2016 Oct 7;12(10):e1006345. doi: 10.1371/journal.pgen.1006345 (PMC5065131; doi:10.1371/journal.pgen.1006345)
Supplement: S2 Fig — (PDF) [file pgen.1006345.s002.pdf]

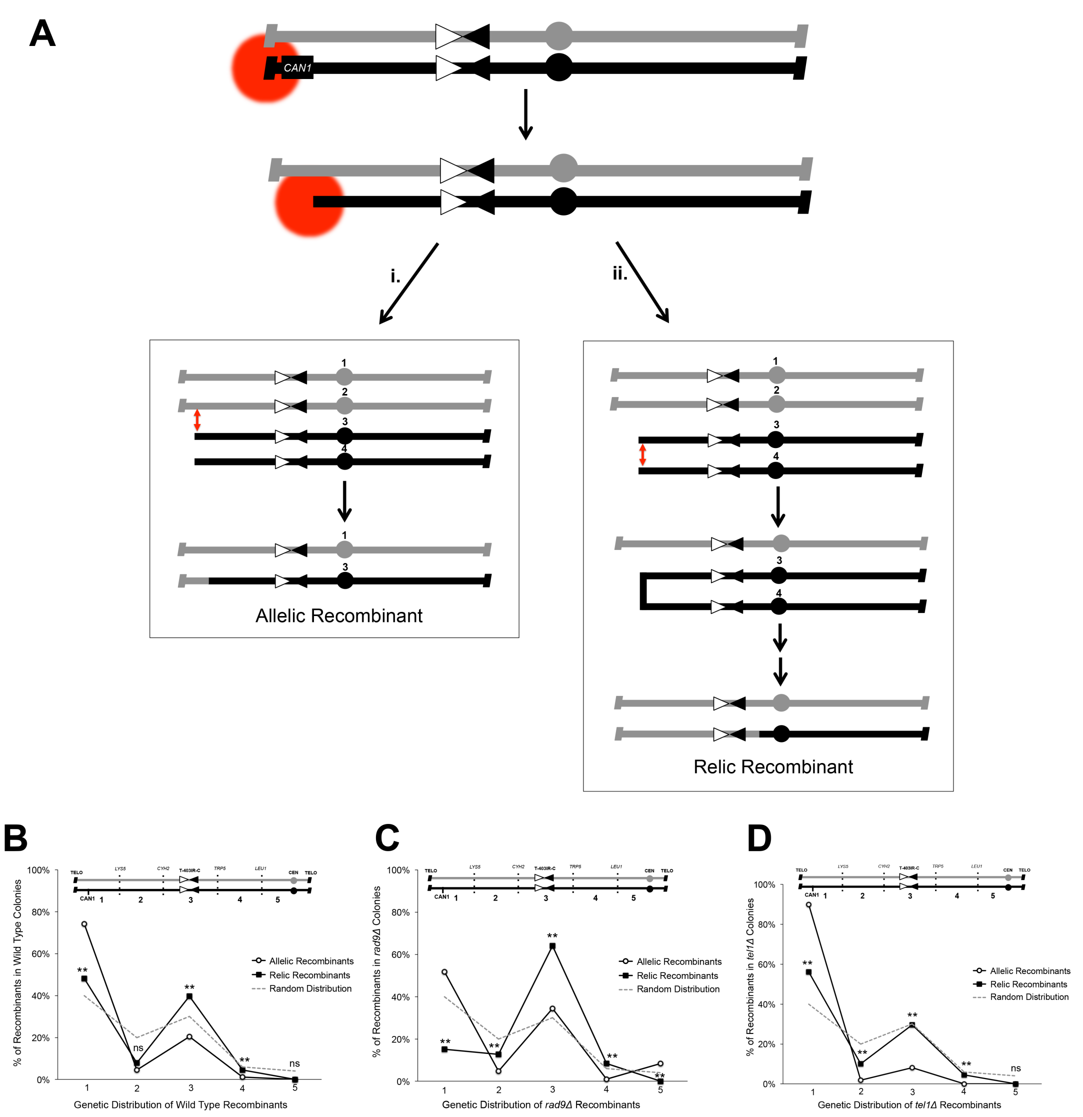

S2 Fig. Distinct ontogenies of allelic and relic recombinants. (A) An initial error or lesion (red circle) forms either an allelic recombinant after repair with the homolog (option i.; red arrow indicates interaction between Chr VII homologs), or an unstable chromosome after repair with the sister chromatid (option ii.; red arrow indicates interaction between sister chromatids), that generates relic recombinants after subsequent damage and repair with the homolog. See Figure 1 legend for symbol key. **(B, C, and D)** Genetic distributions of allelic recombinants (wild type N=397, *rad9Δ* N=420, and *tel1Δ* N=475) and relic recombinants (wild type N=154, *rad9Δ* N=387, and *tel1Δ* N=200) in specific genetic intervals from Can^R Ade$^+$ round and sectored colonies,

respectively. The expected distribution of random allelic recombination is plotted (dashed gray line). Statistically significant differences between allelic and relic recombinant distributions are shown above each genetic interval (*$P < 0.05$, **$P < 0.01$, or non-significant (ns), Z score test for population proportions). Recombinant distributions of wild type, *rad9Δ*, and *tel1Δ* cells are from controls (6hr growth on rich media followed by Can^R Ade^+ selection) of randomized replication stress experiments (treatment of cells with HU and MMS) presented in Fig 2 (*rad9Δ*) and S3 Fig (wild type and *rad9Δ*) and S7 Fig (*tel1Δ*).

Allelic and relic recombinants amongst Can^R Ade^+ round and sectored colony populations, respectively, show distributions that are distinct from the expected random distributions (dashed gray line). Interestingly, for wild type, *rad9Δ*, and *tel1Δ* cells, allelic recombinants tend to be enriched near the chromosome end (genetic interval 1), relative to relic recombinants of each, which are instead enriched near the T-403IR-C region in the middle of the chromosome (genetic interval 3; S2B-D). We propose that a common initial error, a telomere error, either undergoes recombination with the homolog to form an allelic recombinant near the chromosome end or generates an unstable chromosome that later recombines with the homolog along an internal region to form a relic recombinant (diagram of model in S2A).
