# Supplementary material for: Ontogeny of Unstable Chromosomes Generated by Telomere Error in Budding Yeast
Source: PLoS Genet. 2016 Oct 7;12(10):e1006345. doi: 10.1371/journal.pgen.1006345 (PMC5065131; doi:10.1371/journal.pgen.1006345)
Supplement: S3 Fig — (PDF) [file pgen.1006345.s003.pdf]

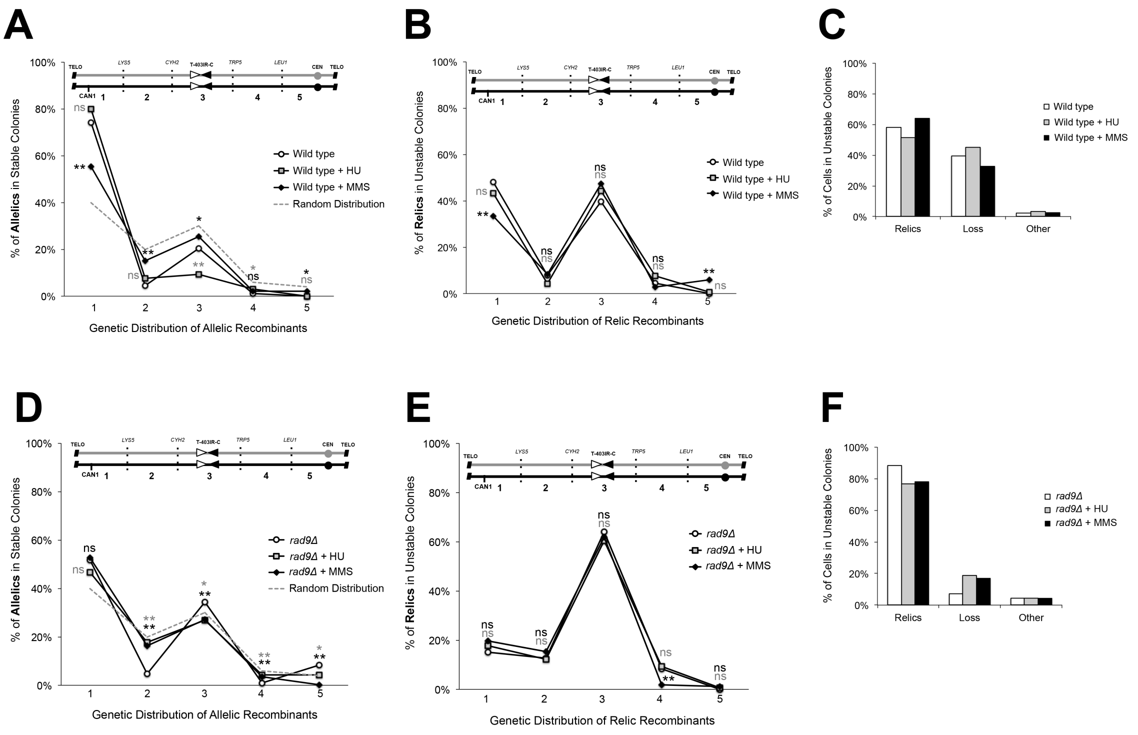


**S3 Fig. Recombinant genetic distributions in wild type and *rad9Δ* are unchanged after random damage.** **(A, D)** Genetic distributions of allelic recombinants in cells from Can$^R$ Ade$^+$ stable colonies after either no treatment (wild type N=397, *rad9Δ* N=420), or a 6 hr HU exposure (wild type N=459, *rad9Δ* N=374), or a 6 hr MMS exposure (wild type N=427, *rad9Δ* N=476). The expected distribution of random allelic recombination is plotted (dashed gray line). **(B, E)** Genetic distributions of relic recombinants in cells from Can$^R$ Ade$^+$ unstable colonies after either no treatment (wild type N=154, *rad9Δ* N=387), or a 6 hr HU exposure (wild type N=169, *rad9Δ* N=379), or a 6 hr MMS exposure (wild type N=255, *rad9Δ* N=260). Statistically significant differences between no treatment control and either 6 hr HU (gray) or MMS (black) exposures are shown above each genetic interval (*P < 0.05, **P < 0.01, or non-significant (ns), Z score test for population proportions). **(C, F)** Distributions of relic recombinants, loss, and "other" from wild type or *rad9Δ* Can$^R$ Ade$^+$ sectored colonies (wild type: untreated N=265, HU exposure N=397, MMS exposure N=397, *rad9Δ*: untreated N=438, HU exposure N=493, MMS exposure N=332).

The altered wild type and *rad9Δ* allelic recombinant distributions after MMS exposure (and *rad9Δ* after HU exposure) are consistent with increasingly random sites of initial errors upon drug exposure (i.e. recombination in each genetic interval is more similar to the expected random distribution after drug treatment). In contrast, Wild Type + HU allelic recombinant distributions are further enriched at the chromosome end and decreased internally relative to no treatment controls, suggesting that HU induces initial events telomere-proximally in wild type cells. Interestingly, though allelic recombinant distributions are generally more random after drug treatment, enrichment of recombination at the chromosome end, relative to the random expectation, remains. The

allelic recombinant distributions after HU and MMS exposures suggests that internal errors are induced, yet the chromosome end continues to undergo recombination most frequently (also see S7 Fig for *tel1Δ* recombinants after HU or MMS exposures). HU and MMS exposure induces unstable chromosomes in wild type and *rad9Δ* (see Fig 2C), but surprisingly the genetic distributions of relic recombinants remain largely unchanged. The unchanged distribution of relic recombinants after random damage suggests either that unstable chromosomes initiate randomly and then recover to the T-403IR-C region or that even random damage initiates instability in the T-403IR-C region (though allelics are not enriched in the region; see Discussion). We propose that the T-403IR-C region is a “collection site”, as opposed to a fragile site where events begin.
