# Supplementary material for: Ontogeny of Unstable Chromosomes Generated by Telomere Error in Budding Yeast
Source: PLoS Genet. 2016 Oct 7;12(10):e1006345. doi: 10.1371/journal.pgen.1006345 (PMC5065131; doi:10.1371/journal.pgen.1006345)
Supplement: S4 Fig — (PDF) [file pgen.1006345.s004.pdf]

A

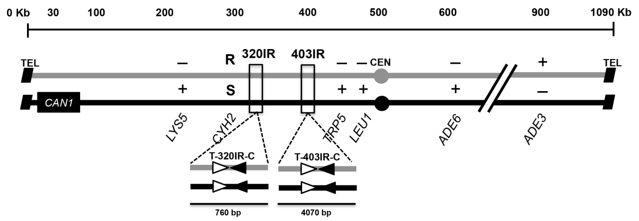

B

Strain	Frequency Allelic Recombination (x 10^{-5})	Frequency Unstable Chromosomes (x 10^{-5})	Frequency Chromosome Loss (x 10^{-5})
Wild type	14 ± 14.5 (1.0)	5.8 ± 3.8 (1.0)	33 ± 26 (1.0)
Wild type *T-403IR-CΔ*	6.8 ± 2.6 (0.49)	**2.75 ± 0.8 (0.47)***	**13 ± 6.6 (0.39)***
rad9Δ	15 ± 12 (1.0)	91 ± 54.5 (1.0)	550 ± 800 (1.0)
rad9Δ T-320IR-CΔ	**5.5 ± 2.8 (0.37)***	54 ± 11 (0.59)	730 ± 1000 (1.3)
rad9Δ T-403IR-CΔ	19 ± 36 (1.3)	76 ± 33.5 (0.83)	550 ± 310 (1.0)
rad9Δ T-320IR-CΔ T-403IR-CΔ	**6.1 ± 3.9 (0.41)***	**39 ± 8.5 (0.43)****	360 ± 250 (0.65)
rad17Δ	130 ± 68 (1.0)	580 ± 140 (1.0)	880 ± 1050 (1.0)
rad17Δ T-403IR-CΔ	**97 ± 100 (0.75)***	**350 ± 140 (0.60)****	840± 880 (0.95)
rad18Δ	82 ± 27 (1.0)	310 ± 81 (1.0)	1000 ± 1200 (1.0)
rad18Δ T-403IR-CΔ	**38 ± 20 (0.46)***	**170 ± 36 (0.55)****	830 ± 550 (0.83)
rad51Δ	4.3 ± 2.5 (1.0)	260 ± 14 (1.0)	1700 ± 1550 (1.0)
rad51Δ T-403IR-CΔ	6.7 ± 2.2 (1.6)	220 ± 88 (0.85)	770 ± 710 (0.45)

C

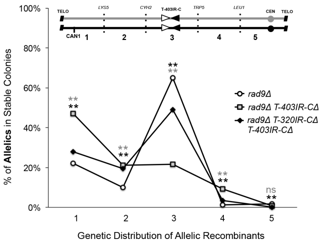

D

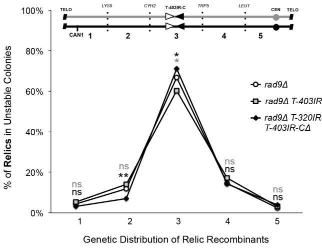

E

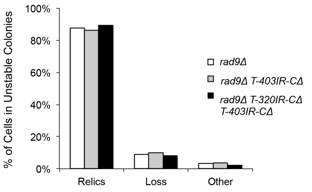

F

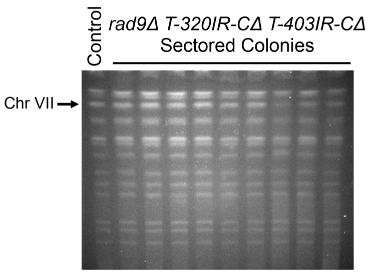

G

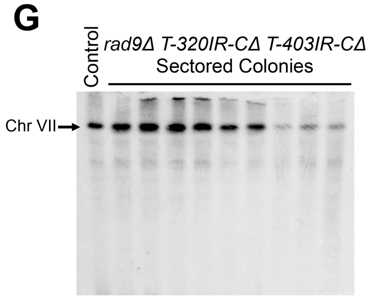

S4 Fig. Unstable chromosomes form in the absence of T-403IR-C site with its tRNA genes and inverted repeats. **(A)** The T-403IR-C inverted repeats (LTR σ sequences, S2 and S3), and/or the T-320IR-C (LTR δ sequences, D4 and D5) inverted repeats, were deleted from both Chr VII homologs (deletion sizes indicated). In addition to the LTR sequences, the 4069 bp deletion in the T-403IR-C site removed two tRNA genes, *PRM8*, *MST27*, and two LTR δ sequences (D7 and D9). **(B)** Frequency of three

instability events in inverted repeat deletion strains relative to controls with intact inverted repeats. Average frequency ± standard deviation shown. Fold changes are in parentheses and are relative to same strain with intact inverted repeats. Statistically significant differences are in bold (*P value < 0.05, **P value < 0.01, Kruskal Wallis test). **(C)** Genetic distributions of allelic recombinants in specific genetic intervals from Can^R Ade$^+$ sectored colonies from *rad9Δ* (N=837), *rad9Δ T-403IR-CΔ* (N=609), and *rad9Δ T-320IR-CΔ T-403IR-CΔ* (N=878). **(D)** Genetic distributions of relic recombinants in specific genetic intervals from Can^R Ade$^+$ sectored colonies from *rad9Δ* (N=994), *rad9Δ T-403IR-CΔ* (N=384), and *rad9Δ T-320IR-CΔ T-403IR-CΔ* (N=532). Statistically significant differences between *rad9Δ* and either *rad9Δ T-403IR-CΔ* (gray) or *rad9Δ T-320IR-CΔ T-403IR-CΔ* (black) exposures are shown above each genetic interval (*P < 0.05, **P < 0.01, or non-significant (ns), Z score test for population proportions). **(E)** Distributions of relic recombinants, loss, or "other" recovered from Can^R Ade$^+$ sectored colonies from *rad9Δ* (N=1134), *rad9Δ T-403IR-CΔ* (N=445), and *rad9Δ T-320IR-CΔ T-403IR-CΔ* (N=594). Relics: relic recombinants, Loss: chromosome loss, Other: complex genotypes (i.e.: half of the cells in a colony have lost the chromosome and the other half retain a relic recombinant.) **(F)** Pulsed field gel stained with ethidium bromide. Individual Can^R Ade$^+$ sectored colonies (unstable colonies) were grown from *rad9Δ T-320IR-CΔ T-403IR-CΔ.* Control: *rad9Δ T-320IR-CΔ T-403IR-CΔ* stock cells. **(G)** Southern blot of pulsed field gel in S4F Fig using a probe to the Chr VII centromere (Chr VII 500 Kb).

The T-403IR-C Site: A collection site, rather than a fragile site

The T-403IR-C site contains a number of sequence features that suggest it is a site of initial instability (Figure 1A). Opposing tRNA genes stall replication forks that converge on this site [1]; this is a known termination site [2]; the region contains a number of DNA fragments linked to DNA breaks and their subsequent repair [3]; and this site shows evidence of translocations and hyper recombination throughout evolution [4]. Additionally, the T-403IR-C site shows increased Rad51 association [5].

The enrichment of unstable chromosome relics in the T-403IR-C region suggested that unstable chromosomes formed in the region. We asked, what is the role of the 403 inverted repeats, if any, in the formation and propagation of unstable chromosomes? We deleted the inverted repeats and tRNA genes from both Chr VII homologs (*T-403IR-CΔ*) and measured all forms of instability and in a variety cells (wild type, *rad9Δ*, *rad17Δ*, *rad18Δ*, and *rad51Δ*; Figure 2C and S4B). We found that the frequencies of unstable chromosomes were unchanged in two strains (*rad9*Δ and *rad51Δ*), and suppressed by, at most, two-fold relative to strains with the 403 Kb inverted repeats in three strains (wild type, *rad17Δ*, and *rad18Δ*). Two-fold changes are statistically significant, though we find empirically that two-fold changes are at the lower limit of interpretation. Additionally, in *rad9*Δ cells we deleted another pair of inverted repeats, present 320 Kb from the telomere, from both Chr VII homologs (*T-320IR-CΔ*), and again found a two-fold reduction in instability. Finally, we evaluated recombinants from cells deleted for the inverted repeats. We found that *rad9Δ IRΔ* allelic distributions were significantly altered while relic recombinants were generally unchanged (S4C and S4D Figs). Allelic recombinants in *rad9Δ IRΔ* cells had 16% (*rad9Δ T-320IR-CΔ T-403IR-CΔ*) and 43%

(*rad9Δ T-403IR-CΔ*) fewer recombinants within the T-403IR-C region (genetic interval 3) relative to *rad9Δ* IR$^+$ cells, suggesting the sites are hotspots for allelic recombination. The distribution of *rad9Δ IRΔ* relic recombinants from either *rad9Δ IRΔ* strain were similar to the distribution from *rad9Δ* (S4D Fig). We also failed to identify any new relic translocations that may have provided clues to mechanism (S4F and S4G Figs). We conclude that the T-403IR-C inverted repeats are not required to form unstable chromosomes. And, though some instability can initiate in the region, deletion of the T-403IR-C inverted repeats does not affect the ability of this region to serve as a "collection site" for events initiated telomere-proximally. The “collection site” interpretation is supported by the MMS and HU treatment of wild type, *rad9Δ, tel1Δ* and telomerase mutant strains, where random or telomere error generates unstable chromosomes that still harbor relics enriched in the T-403IR-C region. What feature(s) of this region predisposes it to "collection" remains unclear.

References:

1. Admire A, Shanks L, Danzl N, Wang M, Weier U, Stevens W, et al. Cycles of chromosome instability are associated with a fragile site and are increased by defects in DNA replication and checkpoint controls in yeast. Genes Dev. 2006;20: 159–173. doi:10.1101/gad.1392506
2. Fachinetti D, Bermejo R, Cocito A, Minardi S, Katou Y, Kanoh Y, et al. Replication Termination at Eukaryotic Chromosomes Is Mediated by Top2 and Occurs at Genomic Loci Containing Pausing Elements. Mol Cell. 2010;39: 595–605. doi:10.1016/j.molcel.2010.07.024
3. Yu X, Gabriel A. Patching broken chromosomes with extranuclear cellular DNA. Mol Cell. 1999;4: 873–881. doi:10.1016/S1097-2765(00)80397-4
4. Kellis M, Patterson N, Endrizzi M, Birren B, Lander ES. Sequencing and comparison of yeast species to identify genes and regulatory elements. Nature. 2003;423: 241–54. doi:10.1038/nature01644
5. Szilard RK, Jacques P-E, Laramée L, Cheng B, Galicia S, Bataille AR, et al. Systematic identification of fragile sites via genome-wide location analysis of gamma-H2AX. Nat Struct Mol Biol. 2010;17: 299–305. doi:10.1038/nsmb.1754
