# Supplementary material for: Ontogeny of Unstable Chromosomes Generated by Telomere Error in Budding Yeast
Source: PLoS Genet. 2016 Oct 7;12(10):e1006345. doi: 10.1371/journal.pgen.1006345 (PMC5065131; doi:10.1371/journal.pgen.1006345)
Supplement: S5 Fig — (PDF) [file pgen.1006345.s005.pdf]

A

Cells	Frequency Allelic Recombination (x 10^{-5})	Frequency Unstable Chromosomes (x 10^{-5})	Frequency Chromosome Loss (x 10^{-5})
EP Vector	16 ± 4.3 (1.0)	59 ± 11 (1.0)	98 ± 27 (1.0)
EP ADH-EST3	13 ± 2.1 (0.84)	**43 ± 5.5 (0.73)***	110 ± 17 (1.1)
EP ADH-Est3-R110A	**31 ± 19 (1.9)***	**110 ± 51 (4.2)***	**190 ± 38 (3.1)****
LP Vector	10 ± 4.3 (1.0)	25 ± 7.0 (1.0)	62 ± 7.1 (1.0)
LP ADH-EST3	14 ± 5.1 (1.4)	27 ± 7.2 (1.1)	84 ± 28 (1.4)*
LP ADH-Est3-R110A	**43 ± 56 (4.3)***	**170 ± 155 (6.7)****	**185 ± 100 (3.0)****

B

Cells	Frequency Allelic Recombination (x 10^{-5})	Frequency Unstable Chromosomes (x 10^{-5})	Frequency Chromosome Loss (x 10^{-5})
EP ADH-Est1-K444E	14 ± 12 (1.0)	79 ± 79 (1.0)	96 ± 21 (1.0)
LP ADH-Est1-K444E	**51 ± 44 (3.6)***	**250 ± 160 (3.2)***	**240 ± 93 (2.5)****
EP ADH-Est3-R110A	31 ± 19 (1.0)	110 ± 51 (1.0)	190 ± 38 (1.0)
LP ADH-Est3-R110A	43 ± 56 (1.4)	170 ± 155 (1.6)	185 ± 100 (0.95)

C

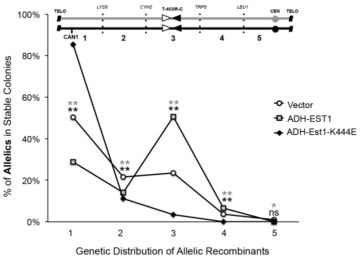

D

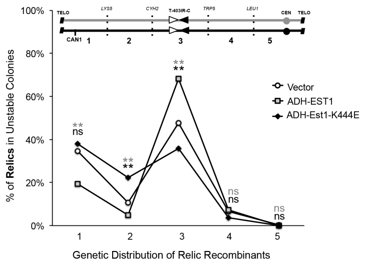

E

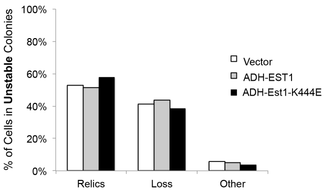

F

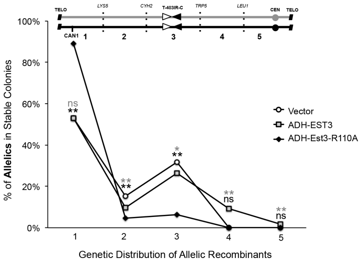

G

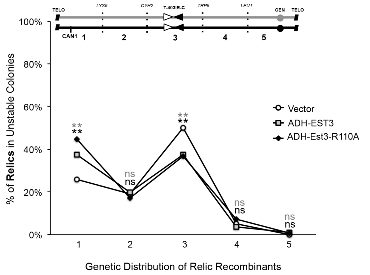

H

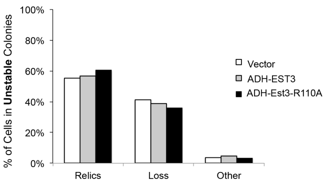

I

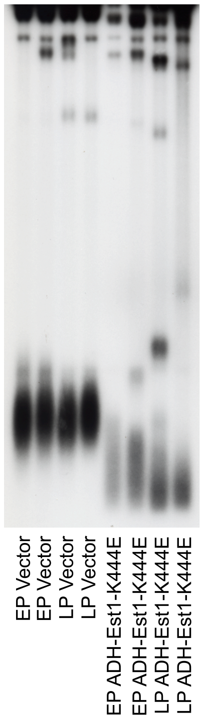

J

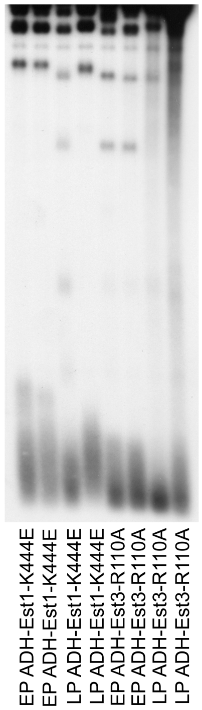

K

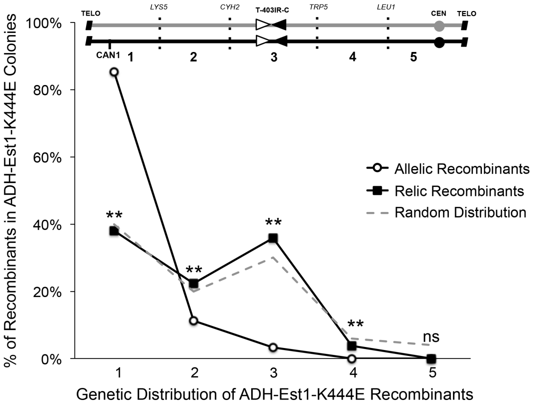

L

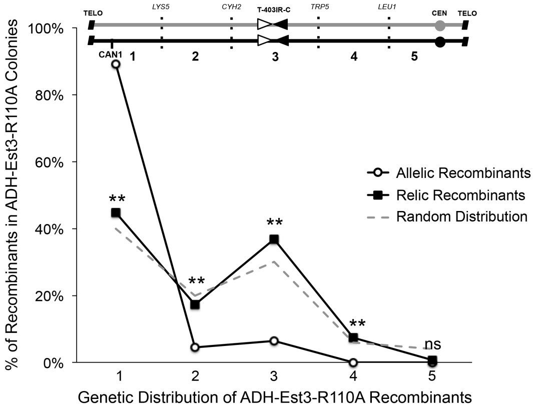

S5 Fig. Unstable chromosomes are induced by telomerase defects. **(A)** Telomerase mutants generate all three forms of instability (allelic recombinants, unstable chromosomes, and loss). Average frequency ± standard deviation shown. Fold changes, relative to vector and within respective passage group, are shown in parentheses. Statistically significant differences are in bold (*P value < 0.05, **P value < 0.01, Kruskal

Wallis test); EP: early passage cells, LP: late passage cells. **(B)** Frequency of instability events comparing the two telomerase defective strains (Fold changes and statistical tests are between EP and LP cells of each strain (*P value < 0.05, **P value < 0.01, Kruskal Wallis test). **(C, F)** Genetic distributions of allelic recombinants in specific genetic intervals from late passage cells expressing EST1 alleles (Vector: N=775, ADH-EST1: N=700, ADH-Est1-K444E: N=648) or EST3 alleles (Vector: N=580, ADH-EST3: N=547, ADH-Est3-R110A: N=788). **(D, G)** Genetic distributions of relic recombinants in specific genetic intervals from late passage cells expressing EST1 alleles (Vector: N=291, ADH-EST1: N=287, ADH-Est1-K444E: N=373) or EST3 alleles (Vector: N=310, ADH-EST3: N=372, ADH-Est3-R110A: N=437). Statistically significant differences between vector and either ADH-EST (gray) or ADH-Est (black) relic recombinants are shown above each genetic interval (*P < 0.05, **P < 0.01, or non-significant (ns), Z score test for population proportions). **(E and H)** Distributions of relic recombinants, loss, or "other" recovered from Can^R Ade^+ sectored colonies from late passage cells expressing EST1 (Vector: N=549, ADH-EST1: N=559, ADH-Est1-K444E: N=645) or EST3 (Vector: N=562, ADH-EST3: N=656, ADH-Est3-R110A: N=722) alleles. **(I)** Native telomere length analysis. Southern blot analysis of XhoI-digested DNA from exponentially growing cells expressing either early passaged (EP) or late passaged (LP) Est1 telomerase dominant negative alleles, or vector controls, using a poly(GT) telomere-specific probe. Two independent isolates of each strain were analyzed. **(J)** Native telomere length analysis. Southern blot analysis of XhoI-digested DNA from exponentially growing cells expressing either EP or LP cells of Est1 or Est3 telomerase dominant negative alleles, using a poly(GT) telomere-specific probe. Two independent isolates of each strain were analyzed. **(K, L)** Genetic distributions of allelic and relic recombinants in specific genetic intervals from late passage cells expressing Est alleles (same allelic and relic Est populations as displayed in C, D, F and G). Statistically significant differences between allelics and relics are shown above each genetic interval (*P < 0.05, **P < 0.01, or non-significant (ns), Z score test for population proportions).

Similar to overexpression of an Est1 dominant negative allele shown in Fig 3, overexpression of the Est3 dominant negative mutant allele significantly increased the frequency of instability (S5A Fig). The frequency of unstable chromosomes formed in late passages of cells expressing ADH-Est3-R110A, relative to early passage, was not significantly increased (S5B Fig); we attribute this modest increase in instability between early and late cell passages to the already substantial increase in unstable chromosomes. We note that telomere lengths of cells expressing ADH-Est3-R110A show exaggerated telomere shortening relative to ADH-Est1-K444 within early passages (S5J Fig; the ADH-Est3-R110A has a stronger instability phenotype than the ADH-Est1-K444E allele, showing an ~2 fold increase in unstable chromosomes within 3 cell passages.)

The increased frequency of unstable chromosomes formed in late passages, relative to early passages, of cells expressing the ADH-Est1-K444E allele is consistent with increased instability correlating with shorter telomeres (S5B Fig). The relative contributions of the absence of telomerase and shorter telomeres to instability remain difficult to determine (see Discussion on the role of telomerase). Native telomere lengths in cells overexpressing either the ADH-Est3-R110A alleles also show an expected decrease in telomere length ([1] and S5J Fig).

Recombinants from cells expressing the telomerase alleles were evaluated. We found that allelic recombinants from stable colonies were enriched near the chromosome end for cells expressing either Est1 or Est3 dominant negative alleles. Relic recombinants from unstable colonies from cells expressing either telomerase dominant negative allele showed a significant shift in genetic interval distribution, increasing the percentage of relics recovered within the T-403IR-C region (S5K and S5L Figs). This result suggests that initial events forming in or near the telomere might progress towards the middle of the chromosome.

References:

1. Lubin JW, Rao T, Mandel EK, Wuttke DS, Lundblad V. Dissecting protein function: An efficient protocol for identifying separation-of-function mutations that encode structurally stable proteins. Genetics. 2013;193: 715–725. doi:10.1534/genetics.112.147801
