# Supplementary material for: Ontogeny of Unstable Chromosomes Generated by Telomere Error in Budding Yeast
Source: PLoS Genet. 2016 Oct 7;12(10):e1006345. doi: 10.1371/journal.pgen.1006345 (PMC5065131; doi:10.1371/journal.pgen.1006345)
Supplement: S6 Fig — (PDF) [file pgen.1006345.s006.pdf]

A

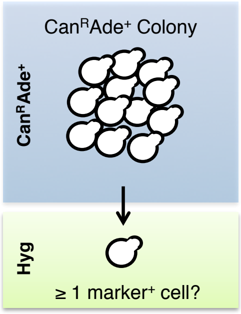

Genetic Marker	% Sectored Colonies Retaining Gene (n = 272)	% Round Colonies Retaining Gene (n = 90)	Expected % Colonies Retaining Gene
HPH	16.5%	8.9%	15%
KANMX	19.1%	11.1%	24%
LYS5	26.5%	35.6%	44%
NAT	50.7%	42.2%	56%
LEU1	99.6%	100%	96%

B

**Cells**	**Frequency Allelic Recombination ($x 10^{-5}$)**	**Frequency Unstable Chromosomes ($x 10^{-5}$)**
rad9Δ Can^R Ade^+	12 ± 6.25 (1.0)	69 ± 16 (1.0)
rad9Δ Can^R Ade^+ Hyg^R	**1.1 ± 0.6 (0.09)****	**5.9 ± 1.25 (0.08)****

S6 Fig. Longer Unstable Chromosomes are Highly Unstable. **(A)** Telomeric genetic markers are rapidly lost from longer unstable chromosomes. Can^R Ade^+ colonies, sectored and round, from *rad9*Δ cells carrying the extensively marked Chr VII (see Figure 4A) were analyzed for retention of telomeric genetic markers. Percentages of cells retaining genetic markers from each population are shown. The expected percentage* of colonies retaining genetic markers was calculated based on genetic interval sizes (distances between genetic markers). **(B)** Frequency of instability events in *rad9*Δ cells carrying the extensively marked Chr VII (see Figure 4A). Fold changes and statistical significance between Can^R Ade^+ Hyg^R colonies relative to Can^R Ade^+ colonies (**P value < 0.01, Kruskal Wallis test).

If an initial unstable chromosome forms in or near the telomere, we expect that genetic markers near the telomere would be retained within unstable colonies. To test for the presence of telomere proximal genetic markers in unstable colonies, we plated cells from unstable colonies to each selective marker. To our surprise, unstable colonies retained telomere proximal genetic markers nearly as often as did stable colonies, and the percent of colonies retaining telomere proximal genetic markers was similar to the expected distribution*. We then tested if the longer unstable chromosomes might be formed, but are rapidly lost or converted to other forms (see Figure 4).

Under selection for telomeric markers (Hyg^R), we found that fewer macrocolonies form (Figure 4B). Figure S6B shows the frequency of stable and unstable colonies in the increased selection. We found that the decreased frequency of macrocolonies under Hyg^R selection correlated with an increase in the frequency of microcolonies. Our results suggest that the microcolonies represent cells containing longer unstable chromosomes that are undergoing loss of the drug resistant selective markers, and subsequently arresting on the increased selection plates.

*Example of calculating expected gene retentions: the HPH gene is located ~75 Kb from the telomere along an ~500 Kb chromosome arm. It is expected that ~15% (75 Kb/ 500 Kb) of colonies would retain the HPH gene.
