# Supplementary material for: Ontogeny of Unstable Chromosomes Generated by Telomere Error in Budding Yeast
Source: PLoS Genet. 2016 Oct 7;12(10):e1006345. doi: 10.1371/journal.pgen.1006345 (PMC5065131; doi:10.1371/journal.pgen.1006345)
Supplement: S7 Fig — (PDF) [file pgen.1006345.s007.pdf]

A

**Cells**	**Frequency Allelic Recombination ($x10^{-5}$)**	**Frequency Unstable Chromosomes ($x10^{-5}$)**	**Frequency Chromosome Loss ($x10^{-5}$)**
Vector	11 ± 5.9 (1.0)	36 ± 10 (1.0)	91 ± 21 (1.0)
rrm3Δ + Vector	**37 ± 5.0 (3.4)****	**79 ± 26 (2.2)***	**190 ± 25 (2.1)****
ADH-Est3-R110A	130 ± 150 (1.0)	245 ± 160 (1.0)	250 ± 140 (1.0)
rrm3Δ + ADH-Est3-R110A	150 ± 130 (1.1)	330 ± 350 (1.3)	**890 ± 620 (3.6)****

B

**Cells**	**Frequency Allelic Recombination ($x10^{-5}$)**	**Frequency Unstable Chromosomes ($x10^{-5}$)**	**Frequency Chromosome Loss ($x10^{-5}$)**
tel1Δ	92 ± 21 (1.0)	180 ± 56 (1.0)	260 ± 87 (1.0)
tel1Δ + HU	**170 ± 41 (1.8)****	**710 ± 120 (3.9)****	**1200 ± 290 (4.6)****
tel1Δ + MMS	**500 ± 100 (5.4)****	**780 ± 140 (4.3)****	**1800 ± 330 (6.9)****

C

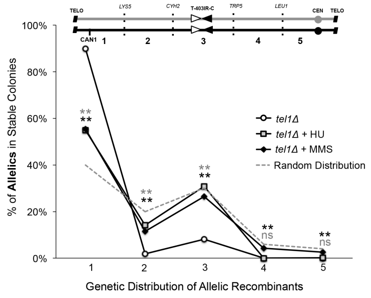

D

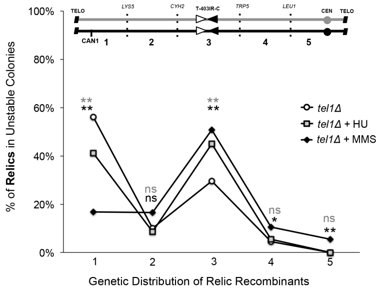

E

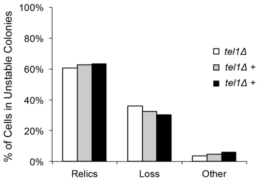

S7 Fig. Telomere dysfunction and replication defects induce unstable chromosomes. (A) Frequency of three instability events in late passage *rrm3Δ* cells expressing Est3 alleles. Average frequency ± standard deviation is shown. Fold changes relative to wild type cells expressing Est3 alleles are shown in parentheses. Statistically significant differences are in bold (*P value < 0.05, **P value < 0.01, Kruskal Wallis test). **(B)** Frequency of three instability events in *tel1Δ* mutants. Average frequency ± standard deviation is shown. Fold changes are in parentheses and are relative to same strain without drug treatment. Statistically significant differences are in bold (**P value < 0.01, Kruskal Wallis test). **(C)** Genetic distributions of *tel1Δ* allelic recombinants in cells from Can^R Ade^+ stable colonies after either no treatment (N=475), or a 6 hr HU exposure

(N=442), or a 6 hr MMS exposure (N=482). The expected distribution of random allelic recombination is plotted (dashed gray line). **(D)** Genetic distributions of *tel1Δ* relic recombinants in cells from Can^R Ade^+ unstable colonies after either no treatment (N=200), or a 6 hr HU exposure (N=256), or a 6 hr MMS exposure (N=219). Statistically significant differences between no treatment control and either 6 hr HU (gray) or MMS (black) exposures are shown above each genetic interval (*P < 0.05, **P < 0.01, or non-significant (ns), Z score test for population proportions). **(E)** Distributions of relic recombinants, loss, and "other" from *tel1Δ* Can^R Ade^+ sectored colonies. (untreated N=331, HU exposure N=408, MMS exposure N=345).

Overexpression of the telomerase dominant negative allele, ADH-Est3-R110A, in *rrm3Δ* mutants did not significantly increase the frequency of unstable chromosomes (S7A Fig).

The genetic distributions of recombinants in *tel1Δ rrm3Δ* were not analyzed because of the high frequency of instability in the strain. We find that even the round *tel1Δ rrm3Δ* Can^R Ade^+ colonies have some instability relative to our wild type round Can^R Ade^+ colonies (< 90% of cells from a round *tel1Δ rrm3Δ* Can^R Ade^+ colony have identical phenotypes), and so we are unable to analyze allelic recombinants in the double mutants.

HU and MMS exposures are expected to induce random damage; we found that allelic recombinants are more randomly distributed after HU or MMS exposure, yet a telomeric enrichment persists relative to untreated cells (Fig 2 and S3 Fig). Study of *tel1Δ* mutants further suggests that MMS and HU induce random errors. As shown in Fig 5C, we found that *tel1Δ* mutants generated allelic recombinants predominantly in the telomere-proximal interval. In contrast, MMS- and HU-treated *tel1Δ* cells have an increased frequency of allelic recombinants (and unstable chromosomes), but ~35% fewer recombinants at the chromosome end than untreated cells (allelics are instead distributed more randomly; S7B-E Figs). The increasingly random distribution of allelic recombinants argues that MMS and HU cause chromosome-wide errors. However, we again find that relic recombinants are most often recovered in the T-403IR-C region, suggesting that the T-403IR-C region is serving as a collection site for relic recombinants.
