# Supplementary material for: Ontogeny of Unstable Chromosomes Generated by Telomere Error in Budding Yeast
Source: PLoS Genet. 2016 Oct 7;12(10):e1006345. doi: 10.1371/journal.pgen.1006345 (PMC5065131; doi:10.1371/journal.pgen.1006345)
Supplement: S8 Fig — (PDF) [file pgen.1006345.s008.pdf]

## A

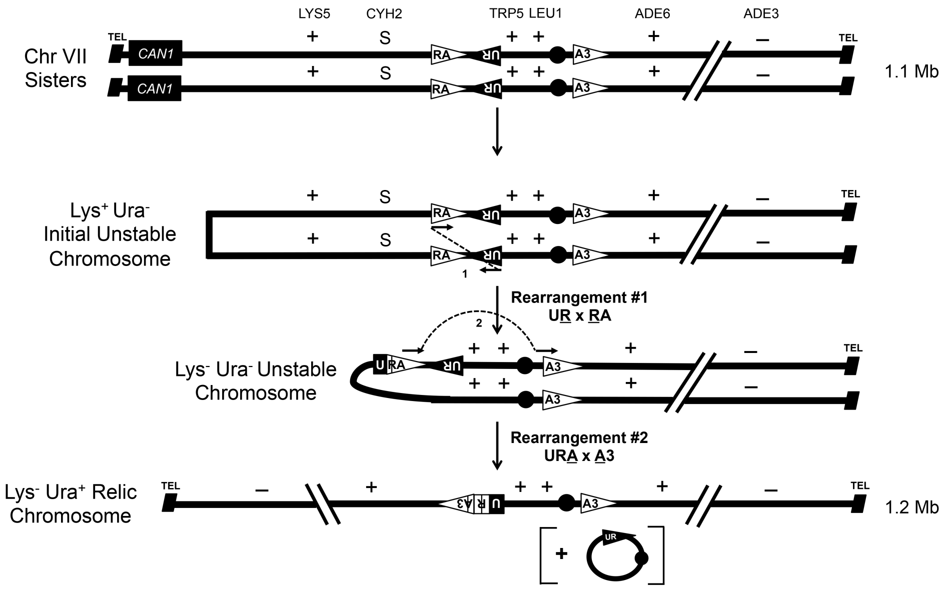


## B

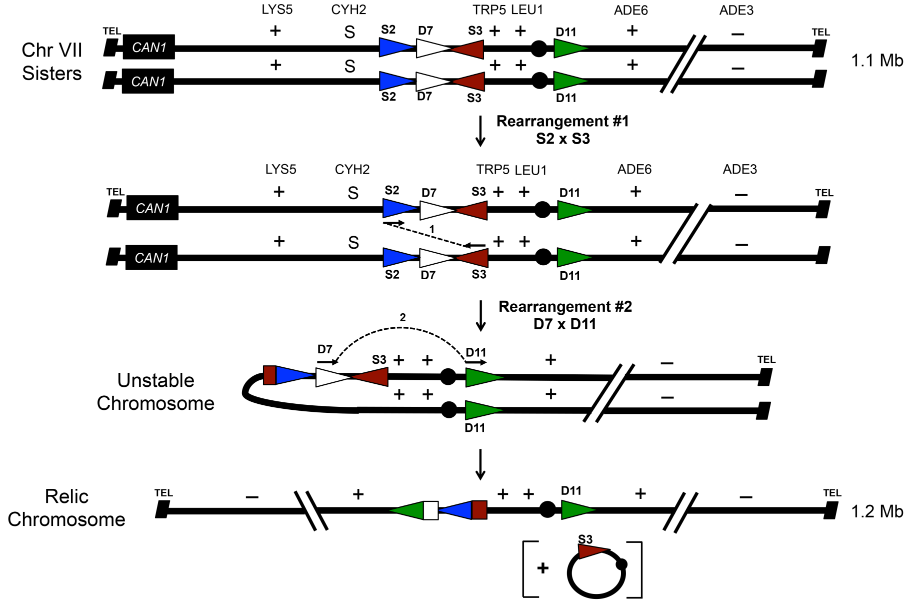


## C

| Cells | Frequency Allelic Recombination (x $10^{-5}$) | Frequency Unstable Chromosomes (x $10^{-5}$) | Frequency Chromosome Loss (x $10^{-5}$) |
|---|---|---|---|
| Wild type UR-RA-A3 | 11 ± 9.9 (1.0) | 6.3 ± 5.2 (1.0) | 44 ± 58 (1.0) |
| *rad9Δ* UR-RA-A3 | 14 ± 7.8 (1.3) | **78 ± 52 (12)**** | **360 ± 410 (8.2)**** |
| *tel1Δ* UR-RA-A3 | **23 ± 14 (2.1)**** | **65 ± 49 (10)**** | **180 ± 135 (4.1)**** |

**D**

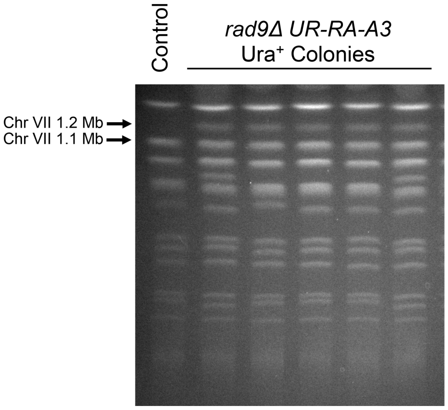


**E**

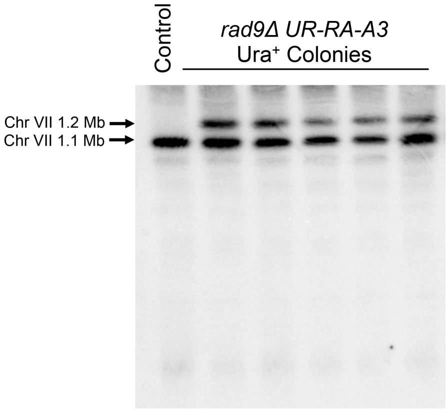


**F**

| **Cells** | **% URA3 Isochromosome in Ura+ Colonies** | **% URA3 Isochromosome Genotype in Ura+ Colonies** |
|---|---|---|
| Wild type UR-RA-A3 | 100% (9/9) | 98.8% (1186/1200) |
| *rad9Δ* UR-RA-A3 | 100% (16/16) | 99.5% (5174/5200) |
| *tel1Δ* UR-RA-A3 | 100% (6/6) | 99.4% (5082/5111) |

**S8 Fig. Longer unstable chromosomes resolve to an isochromosome. (A)** Model of conversion of a long unstable chromosome to a shorter unstable dicentric. The *URA3* gene was divided into three fragments and inserted into the LTR regions of Chr VII as discussed in text and [37]. An initial (or early) fusion between Chr VII sisters with the *CAN1* homolog forms a $Lys^+$ unstable chromosome. The $Lys^+$ unstable chromosome then undergoes Rearrangement #1, a fusion of UR and RA gene fragments (in inverted orientation), to generate a $Lys^-$ dicentric chromosome. The $Lys^-$ URA unstable dicentric then undergoes Rearrangement #2 to form the isochromosome. A circle DNA molecule product is hypothetical. The $Ura^+$ isochromosome was verified by pulsed field gels to be 1.2 Mb in size, larger than the unrearranged 1.1 Mb initial Chr VII (S8D and S8E Figs). **(B)** Model of generation of previously characterized isochromosome. Using the existing chromosome sequences, an unstable dicentric chromosome is formed, which is then converted to a more stable monocentric isochromosome (a relic chromosome; [1]). A fusion between the T-403IR-C inverted repeats, S2 and S3 LTR σ sequences, forms an unstable dicentric chromosome, detected by PCR. A second rearrangement between the D7 and D11 LTR δ sequences removes one centromere to form the isochromosome, also detected by a different PCR reaction. The *URA3* module in Fig 6 and S8A Fig serves as a proxy for the S2/S3 and D7/D11 chromosome rearrangements. **(C)** Frequency of instability events in wild type, *rad9Δ*, and *tel1Δ* cells with integrated URA3 modules (strains TY588, TY416, TY589, respectively). Average frequency ± standard deviation shown. Fold changes are in parentheses and are relative to wild type.

Statistically significant differences are in bold (**P value < 0.01, Kruskal Wallis test). **(D)** Confirmation that a 1.2 MB Ura+ translocation is formed by the dicentric to isochromosome fusion reaction. Ethidium bromide stained pulsed field gel. Control: *rad9Δ UR-RA-A3* stock cells. $Ura^+$ colonies were isolated from $Can^R$ $Ade^+$ sectored colonies. Arrows indicate expected band sizes for unchanged Chr VII (1.1 Mb) or Chr VII URA3 isochromosome (1.2 Mb). **(E)** Southern blot of pulsed field gel in FigS8D using a probe to the Chr VII centromere (Chr VII 500 Kb). Pulsed field gels and southern blots were also performed to verify URA3 translocations in wild type *UR-RA-A3*, and *tel1Δ UR-RA-A3* cells (data not shown). **(F)** Percentages of $Ura^+$ cells carrying the URA3 isochromosome, verified by pulsed field gel electrophoresis and southern blot (as in Figures S8D and S8E), are shown for each strain (column #2). $Ura^+$ cell genotypes were also used to verify the URA3 translocation (column #3). Expected genotype of cells carrying the URA3 translocation: $Lys^-$, $Cyh^R$, $Trp^+$, $Leu^+$, $Ade^+$, $Neo^R$, $Nat^S$ (if the expected DNA circle has been lost from cells). In each strain, over 98% of $Ura^+$ cells displayed the expected genotype. All $Ura^+$ cells that did not have expected genotypes retained $Nat^R$. We predict that these $Ura^+$ cells formed the isochromosome, but may not have yet lost the $Nat^R$ DNA circle. $Ura^+$ colonies were isolated from $Can^R$ $Ade^+$ sectored colonies and then replica plated to selective media to infer genotypes of each (wild type: N=1200 $Ura^+$ colonies isolated from N=12 sectoreds, *rad9Δ*: N=5200 $Ura^+$ colonies isolated from N=7 sectoreds, *tel1Δ*: N=5111 $Ura^+$ colonies isolated from N=6 sectoreds).

The URA3 module serves as a proxy for the rearrangement previously identified in our Chr VII disome [1]. The URA3 isochromosome (or translocation) was previously reported in *rad9Δ* [2]. Here we find that URA3 isochromosomes, formed from a dicentric intermediate, also form in cells with telomere defects, and wild type cells. Further, we infer that a longer, initial unstable chromosome progresses to the dicentric intermediate near the T-403IR-C region and then resolves to a relic recombinant (URA3 isochromosome). The presence of URA3 isochromosome relic recombinants was verified by molecular analysis (pulsed field gel electrophoresis followed by southern blotting with a probe to Chr VII) and genetic analysis. $Ura^+$ cells from wild type, *rad9Δ*, and *tel1Δ* cells all formed the predicted isochromosome at a high frequency (S8 Fig). The few Ura+ cells that did not retain the expected genotype of the isochromosome all retained an unstable $Nat^R$ product; we predict that this $Nat^R$ product is in a DNA circle expected to form during the URA x A3 recombination events (see S8A Fig).

1. Admire A, Shanks L, Danzl N, Wang M, Weier U, Stevens W, et al. Cycles of chromosome instability are associated with a fragile site and are increased by defects in DNA replication and checkpoint controls in yeast. Genes Dev. 2006;20: 159–173. doi:10.1101/gad.1392506
2. Paek AL, Kaochar S, Jones H, Elezaby A, Shanks L, Weinert T. Fusion of nearby inverted repeats by a replication-based mechanism leads to formation of dicentric and acentric chromosomes that cause genome instability in budding yeast. Genes Dev. 2009;23: 2861–2875. doi:10.1101/gad.1862709
