# Supplementary material for: Ontogeny of Unstable Chromosomes Generated by Telomere Error in Budding Yeast
Source: PLoS Genet. 2016 Oct 7;12(10):e1006345. doi: 10.1371/journal.pgen.1006345 (PMC5065131; doi:10.1371/journal.pgen.1006345)
Supplement: S1 Appendix — (PDF) [file pgen.1006345.s009.pdf]

S1 Appendix. Unstable chromosomes were missed in previous studies. The Hackett and Greider, MCB 2003 [1] paper shows the data that suggests to us that they missed unstable chromosomes, a major outcome of telomerase defect; ~80% of telomere error forms unstable chromosomes, ~20% forms stable allelic recombinants in our Chr VII disome. To state explicitly what they did and found: They used a Chr VII diploid, with generally the same genetic markers we used because they use the same Chr VII homolog (except *CAN1* and *ADE5* are near the telomere but not exactly where our *CAN1* is). They grow their diploid cells without telomerase (GAL-EST1 in the off state, in glucose), in liquid media. Events arise and cell death occurs. They then plate cells on canavanine selective media, with galactose to turn back on EST1. Can^R colonies form. They then phenotype the Can^R colonies, and find 90% are recombinants in the telomere-proximal interval (Fig 1E summary of data in [1]).

We suggest that this analysis completely misses unstable chromosomes. In liquid media, when events occur as cells senesce, any Can^R allelic recombinants would quickly overgrow any unstable chromosomes; in our disome unstable chromosome-generated sectored colonies take 4 or 5 days to form, while allelic recombinants take 2 days. This difference may be even more extreme in a diploid, though we do not know that. (A 2N-1 Chr VII monosome grows very poorly compared to a diploid. This is in part why we like the Chr VII disome, as the disome and haploid have comparable growth properties.)

We cannot prove their assay misses unstable chromosomes, but we think it is highly likely.

Reference:

1. Hackett JA, Greider CW. End resection initiates genomic instability in the absence of telomerase. Mol Cell Biol. 2003;23: 8450–61. doi:10.1128/MCB.23.23.8450.
