# Supplementary material for: Ontogeny of Unstable Chromosomes Generated by Telomere Error in Budding Yeast
Source: PLoS Genet. 2016 Oct 7;12(10):e1006345. doi: 10.1371/journal.pgen.1006345 (PMC5065131; doi:10.1371/journal.pgen.1006345)
Supplement: S1 Table — (PDF) [file pgen.1006345.s010.pdf]

S1 Table. *Saccharomyces cerevisiae* strains used in this study

Strain	Genotype[a,b,c,d]	Source
TY200	Wild type	[1]
TY206	*rad9Δ*::ura3	[1]
TY216	*rad17Δ::hisGura3*	[1]
TY339	*rad51Δ::URA3*	[2]
TY416	*rad9Δ::ura3 403::RA::nat1MX::RU 535::A3::KanMX4*	[2]
TY440	*rad18Δ::KanMX4*	[2]
TY580	*rad9Δ::ura3 T-403IR-CΔ::KanMX4, Nat1MX*	This study
TY581	*rad9Δ::ura3 T-320IR-CΔ::URA3, HPH*	This study
TY582	*rad9Δ::ura3 T-320IR-CΔ::URA3, HPH T-403IR-CΔ::Nat1MX, KanMX4*	This study
TY583	*T-403IR-CΔ::KanMX4, Nat1MX*	This study
TY584	*rad17Δ::hisGura3 T-403IR-CΔ::KanMX4, Nat1MX*	This study
TY585	*rad18Δ::KanMX4 T-403IR-CΔ::HPH, Nat1MX*	This study
TY586	*rad51Δ::URA3 T-403IR-CΔ::Nat1MX, KanMX4*	This study
TY587	*rad9Δ::ura3 75::HPH 122::KanMX4 287::nat1MX*	This study
TY588	*403::RA::nat1MX::RU 535::A3::KanMX4*	This study
TY589	*tel1Δ::HPH 403::RA::nat1MX::RU 535::A3::KanMX4*	This study
TY590	*tel1Δ::URA3*	[3]
TY591	*rrm3Δ::KanMX4*	This study
TY592	*tel1Δ::URA3 rrm3::KanMX4*	This study

[a]All strains are disomic for Chr VII and derivatives of TY200 MATa +/hxk2::CAN1 lys5/+ cyh^{r}/CYH^{s} trp5/+ leu1/+ Centromere ade6/+ +/ade3, ura3-2 (A364a genetic background) except for mutations listed [1].
[b]Inverted repeat deletions (*T-320IR-CΔ* or *T-403IR-CΔ*) were removed from both Chr VII homologs and replaced by selective markers. The first selective marker listed was integrated into the *CAN1* Chr VII homolog, and the second was integrated into the other Chr VII homolog.
[c]The URA3 module, or selective markers, were integrated into the *CAN1* Chr VII homolog. Locations of inserts are notated (Kb from the left telomere of Chr VII).
[d]Telomerase defective strains expressed plasmids (S2 Table) in either TY200, TY588, or TY591 background strains.

References

1. Admire A, Shanks L, Danzl N, Wang M, Weier U, Stevens W, et al. Cycles of chromosome instability are associated with a fragile site and are increased by defects in DNA replication and checkpoint controls in yeast. Genes Dev. 2006;20: 159–173. doi:10.1101/gad.1392506
2. Paek AL, Kaochar S, Jones H, Elezaby A, Shanks L, Weinert T. Fusion of nearby inverted repeats by a replication-based mechanism leads to formation of dicentric and acentric chromosomes that cause genome instability in budding yeast. Genes Dev. 2009;23: 2861–2875. doi:10.1101/gad.1862709
3. Kaochar S, Shanks L, Weinert T. Checkpoint genes and Exo1 regulate nearby inverted repeat fusions that form dicentric chromosomes in Saccharomyces cerevisiae. Proc Natl Acad Sci U S A. 2010;107: 21605–10. doi:10.1073/pnas.1001938107
