# Supplementary material for: Ontogeny of Unstable Chromosomes Generated by Telomere Error in Budding Yeast
Source: PLoS Genet. 2016 Oct 7;12(10):e1006345. doi: 10.1371/journal.pgen.1006345 (PMC5065131; doi:10.1371/journal.pgen.1006345)
Supplement: S2 Table — (PDF) [file pgen.1006345.s011.pdf]

S2 Table. Plasmids used in this study

Plasmid	Selective Markers and Alleles	Source
pRS423	2μ *HIS3* plasmid	[1]
pRS426	2μ *URA3* plasmid	[1]
pVL809	2μ *ADH-EST1::HIS3*	V. Lundblad, unpublished
pVL4534	2μ *ADH-Est1-K444E::HIS3*	V. Lundblad, unpublished
pVL895	2μ *ADH-EST3::URA3*	[2,3]
pVL3492	2μ *ADH-Est3-R110A::URA3*	[2,3]

References

1. Christianson TW, Sikorski RS, Dante M, Shero JH, Hieter P. Multifunctional yeast high-copy-number shuttle vectors. Gene. 1992;110: 119–122. doi:10.1016/0378-1119(92)90454-W
2. Lubin JW, Rao T, Mandel EK, Wuttke DS, Lundblad V. Dissecting protein function: An efficient protocol for identifying separation-of-function mutations that encode structurally stable proteins. Genetics. 2013;193: 715–725. doi:10.1534/genetics.112.147801
3. Lee J, Mandell EK, Rao T, Wuttke DS, Lundblad V. Investigating the role of the Est3 protein in yeast telomere replication. Nucleic Acids Res. 2010;38: 2279–2290. doi:10.1093/nar/gkp1173
